# Synthesis of bio-inspired silver nanoparticles using *Geranium wallichianum* D. Don ex Sweet. (Geraniaceae) leaf extract for antibacterial activity and colorimetric detection of Hg^2+^

**DOI:** 10.1186/s11671-026-04673-9

**Published:** 2026-05-29

**Authors:** Attia Khalid, Nasir Assad, Muhammad Naeem-ul-Hassan, Marzia Batool Laila, Dalya Marwan Attallah, Shadi A. Zakai, Khalil Alkuwaity, Yasir Assad, Rao Muhammad Faisal Iqbal, Muhammad Nauman Khan, Alevcan Kaplan, Majid Khan, Sayed Alim Samim

**Affiliations:** 1 Institute of Chemistry, University of Sargodha, Sargodha, 40100 Pakistan; 2 Department of Clinical Microbiology Laboratory, King Abdulaziz University Hospital, Jeddah, Saudi Arabia; 3 Department of Clinical Microbiology and Immunology, Faculty of Medicine, King Abdulaziz University, Jeddah, 21589 Saudi Arabia; 4 Department of Medical Laboratory Sciences, Faculty of Applied Medical Sciences, King Abdulaziz University, Jeddah, 21589 Saudi Arabia; 5 EcoHealth Research Unit, King Fahd Medical Research Center, King Abdulaziz University, Jeddah, 21589 Saudi Arabia; 6 Department of Zoology, Hazara University Mansehra, Mansehra, Khyber Pakhtunkhwa Pakistan; 7 Department of Chemistry, The University of Lahore, Sargodha Campus, 10 km Lahore road, Sargodha, Pakistan; 8 Department of Botany, University of Chakwal, Punjab, 48800 Pakistan; 9 Department of Pharmaceutical Botany, Faculty of Pharmacy, Dicle University, Diyarbakır, 21200 Turkey; 10 Institute of Biotechnology and Genetic Engineering, The University of Agriculture Peshawar, Peshawar, 25130 Pakistan; 11 Faculty of Agriculture, Takhar University, Takhar, 3702 Afghanistan

**Keywords:** Antibacterial activity, Phytochemical capping, Colorimetric detection, Surface plasmon resonance, Green synthesis, *Geranium wallichianum*, Silver nanoparticles

## Abstract

The rise in antimicrobial resistance and heavy metal contamination poses major risks to human health and the environment. This study reports the green synthesis of silver nanoparticles (AgNPs) using a polar extract of *Geranium wallichianum* D. Don ex Sweet. to evaluate antibacterial activity and Hg^2+^-sensing. This is the first reported use of *G. wallichianum* as a dual reducing and stabilizing agent, enabling a rapid sunlight-assisted synthesis completed within 15 min. Characterization confirmed successful formation of AgNPs: UV–Vis spectroscopy monitored stability, FT-IR verified functional group interactions, PXRD confirmed the crystalline phase, SEM revealed an average particle size of 26 nm, and hydrodynamic size and zeta potential were 39 nm and − 28 mV, respectively. Antibacterial activity was assessed using 30 µg/mL AgNPs, 30 µg/mL erythromycin as a positive control, 30 µg/mL plant extract, and DI water as a negative control. All tests were conducted in triplicate and analyzed using one-way ANOVA (*p* < 0.05). The biogenic AgNPs exhibited strong antibacterial activity against *Staphylococcus epidermidis* (20 mm ZOI), *Klebsiella pneumoniae* (18 mm ZOI), and *Escherichia coli* (19 mm ZOI). The MIC and MBC values ranged from 15 to 30 µg/mL, confirming the dose-dependent antibacterial potency. Additionally, AgNPs showed excellent colorimetric response toward Hg^2+^, with a linear detection range of 1–100 µM and a limit of detection (LOD) of 6.68 µM. The sensor demonstrated high selectivity and practical applicability in real-water samples. Overall, the unique sunlight-driven synthesis strategy, coupled with the strong antibacterial potential and exceptional Hg^2^^+^ selectivity, underscores the novelty and sustainability of *G. wallichianum*-mediated AgNPs as a versatile nanoplatform for biomedical and environmental applications.

**Supplementary Information:**

The online version contains supplementary material available at 10.1186/s11671-026-04673-9.

## Introduction

The escalating threat of multidrug-resistant (MDR) bacterial infections is a global public health concern [1]. Each year, approximately 700,000 people worldwide die from bacterial infections resistant to antimicrobial treatments, and this number is estimated to reach nearly 10 million by 2050. The World Health Organization (WHO) has identified a Priority Pathogen List that includes ESKAPE pathogens (*Enterococcus faecium*, *Staphylococcus aureus*, *Klebsiella pneumoniae*, *Acinetobacter baumannii*, *Pseudomonas aeruginosa*, and *Enterobacter* species) because of their high resistance potential and role in hospital-acquired infections. These pathogens often form biofilms, rendering conventional antibiotics ineffective [2]. Therefore, advancements in medicine are needed to combat antibiotic-resistant pathogenic bacteria in the human population [3, 4, 5].

Human health, the environment, and the biosphere are at a high risk of contamination by heavy metals such as lead, cadmium, mercury, and arsenic [6, 7, 8]. Therefore, sensitive and safe systems are required for monitoring and remediating heavy metals [9, 10]. Mercury is extremely dangerous to humans and aquatic life and is considered one of the most hazardous heavy metal ions. Elemental mercury is non-biodegradable and has devastating effects on animals and humans, including DNA damage, inhibition of ligand-receptor binding, liver and kidney damage, immune system dysfunction, and death [11]. Therefore, the accurate detection of mercuric ions (Hg^2+^) is of utmost importance for human health and environmental safety. Silver nanoparticles (AgNPs) are excellent candidates for the colorimetric detection of Hg^2+^ because of their unique plasmonic properties, which enable sensitive and selective detection. AgNPs undergo a color change in the presence of Hg^2^, which has proven to be very effective for real-time, on-site monitoring of mercury contamination. Studies have shown that AgNPs can selectively bind to Hg^2+^, resulting in a specific color change in the solution that is visible to the naked eye. This method provides a rapid and cost-effective solution and improves the sensitivity and specificity of mercury detection, making it a robust tool for protecting human health and maintaining environmental integrity [12].

The most recent developments in the field of nanoparticles (NPs) have provoked a great scientific revolution across all fields, aiming to provide a sustainable environment for humanity [13, 14, 15]. Researchers predominantly used nanodiamondss because of their improved optical, biological, and catalytic properties, as well as their higher surface-to-volume ratio compared to traditional materials [16, 17, 18]. Over the past few decades, nanoparticles have been widely studied for their significant potential to strengthen human immunity against life-threatening diseases, improve energy storage problems, and advance systems for drug and gene delivery [19, 20]. Moreover, nanotechnology is used to advance medical devices, diagnostics, and other therapies. In addition, nanotechnology is making significant contributions to the development of innovative diagnostic methods, medical equipment, and agricultural appliactions [21, 22].

Silver has been used most frequently in medicine due to its antibacterial properties, which are superior to those of other metals such as gold, copper, iron, titanium, and zinc [23, 24]. The antibacterial properties of AgNPs have attracted considerable attention for their potential to inhibit bacterial growth and the development of pathogenic bacteria, including antibiotic-resistant strains [25, 26]. AgNPs can be produced by physical methods, such as mechanical milling, physical vapor deposition, sonication, and laser ablation; however, these methods are costly, require high energy input, and need advanced electrical equipment [27]. Chemical techniques, including the chemical sol-gel process, chemical vapor deposition, and co-precipitation, are not environmentally friendly and involve hazardous materials, such as sodium borohydride, hydrazine, and sodium dodecyl sulfate [25].

The increasing demand for greener and more economically viable methods for synthesizing NPs has led to the development of green synthesis processes [28]. Typically, the green synthesis of AgNPs is achieved using bacteria [29], fungi [30], algae [31], and plants as extracellular or intracellular sources for reducing Ag^+^ to Ag^0^ [32]. Plant-mediated synthesis is the most frequently utilized technique for synthesizing metal NPs [30]. In this technique, various plant parts such as leaves, bark, flowers, fruits, roots, and stems are utilized to generate metal NPs through a process referred to as biofabrication. The secondary metabolites in plant extracts flavonoids, alkaloids, phenols, polyphenols, polyols, and saponins, act as reducing and capping agents. These molecules can modify the properties of the produced NPs and enhance their functionality for different applications [33]. Plants, particularly those with pharmacological properties, are of immense interest and hold great promise in the green synthesis of AgNPs [25]. Nevertheless, *G. wallichianum* is not widely used for NP production, and no studies have explored its unique phytochemical profile for dual reduction and capping activities in AgNP production. This indicates a research gap regarding whether *G. wallichianum* is an efficient and multifunctional biofactory for nanoparticle production. *G. wallichianum*,* also known as* Rattenjot, belongs to the family Geraniaceae [34]. This species is found in the Himalayas, Kashmir, and Nepal at altitudes ranging between 7000 and 11,000 feet. The leaves of the plant possess medicinal value and are used in the treatment of eye diseases, blood cleansing, jaundice, kidney diseases, hepatitis, fever, and leukorrhea [35].

The current study hypothesizes that the phytochemical constituents of *G. wallichianum* can act as both reducing and stabilizing agents under sunlight irradiation to produce stable AgNPs with strong antibacterial activity and highly selective colorimetric sensing capability for Hg^2+^ ions. The synthesized AgNPs were thoroughly characterized using UV-Vis spectroscopy, Fourier-transform infrared (FTIR) spectroscopy, Powder X-ray Diffraction (PXRD), Scanning Electron Microscopy (SEM), Energy-dispersive X-ray (EDX) spectroscopy, and dynamic light scattering (DLS). The antimicrobial activity of the biogenic AgNPs was evaluated against *Staphylococcus epidermidis* (ATCC 14990), *Klebsiella pneumoniae* (ATCC 13883), and *Escherichia coli* (ATCC 11775). Additionally, the synthesized AgNPs were employed as colorimetric sensors for Hg^2+^ detection using water as the solvent. The green synthesis approach utilizing *G. wallichianum* is environmentally friendly, avoids toxic chemicals, and enables sustainable large-scale production. Sunlight-mediated synthesis further enhances cost-effectiveness by reducing energy requirements and utilizing readily available plant resources. The AgNPs demonstrated broad-spectrum antibacterial activity, offering potential for combating antibiotic-resistant pathogens. The synthesized AgNPs also exhibit strong selectivity for mercury ions, making them suitable for environmental monitoring. However, reliance on sunlight may limit reproducibility under variable climatic conditions, and further evaluation of biocompatibility is required for biomedical applications.

## Materials and methods

### Materials

Plant samples were collected from Nathia Gali, Khyber Pakhtunkhwa, Pakistan. The collection and use of *G. wallichianum* in this study complied with all relevant institutional, national, and international guidelines and legislation. The plant was authenticated by Ms. Naima Huma Naveed, Assistant Professor, Department of Botany, Sargodha University, Sargodha, Pakistan, and Dr. Muhammad Nauman Khan, Department of Botany, Islamia College Peshawar, Pakistan. A voucher specimen was deposited under the number SU/CHEM/077 in the herbarium of the Department of Chemistry, University of Sargodha, for future reference. The plant sample (*G. wallichianum*) was collected from privately owned land in Nathia Gali, Khyber Pakhtunkhwa, Pakistan, where it grows naturally. As the collection took place on private property and did not involve any protected or endangered species, no specific permissions or licenses were required.

Silver nitrate (AgNO₃, ≥ 99% purity, Merck, Germany), n-hexane (≥ 95%, EMSURE^®^ ACS, Malaysia), Mueller-Hinton (MH) agar (Bio-Rad, USA), and ethanol (≥ 99.5%, Sigma-Aldrich^®^) were purchased from a local market and were of analytical grade. The reagents used for the phytochemical tests included ferric chloride (≥ 97%, Sigma-Aldrich^®^), sodium hydroxide (≥ 98%, Sigma-Aldrich^®^), lead acetate (≥ 99%, Sigma-Aldrich^®^), and isoamyl alcohol (≥ 98%, Sigma-Aldrich^®^). The interfering compounds used in the study include lead nitrate Pb(NO₃)₂ (≥ 99%, Sigma-Aldrich^®^), cadmium nitrate Cd(NO₃)₂ (≥ 99%, Sigma-Aldrich^®^), nickel nitrate Ni(NO₃)₂ (≥ 98%, Sigma-Aldrich^®^), and cobalt nitrate Co(NO₃)₂ (≥ 98%, Sigma-Aldrich^®^), all of which were purchased from Sigma-Aldrich^®^. The preparation of the solutions and the extractions were performed with deionized water (DI water, resistance of 18.2 MΩ cm).

### Polar extraction from the plant sample

The polar extract from the leaves of *G. wallichianum* was prepared by a conventional method. The leaves were washed with DI water to remove dirt and other impurities, then dried at room temperature for 15 days following the protocol described in [30] with some modifications. The dried leaves were pulverized using a grinder and sieved through a fine sieve with a mesh size of 75 μm. Then, 2.5 g of the fine leaf powder was mixed with 50 mL of DI water, and the solution was stirred for 5 h at room temperature on a stirring plate. The solution was then filtered through Whatman No. 42 paper, and the filtrate was washed with *n*-hexane using a separatory funnel, resulting in two distinct layers: one polar and one non-polar. The nonpolar layer was discarded, and the polar layer was transferred to a Petri dish and dried for 24 h in an air-drying oven at 45 °C. After complete drying, the sample was removed from the Petri dish, stored in the refrigerator, and labeled as *G. wallichianum* extract for further use.

### Phytochemical screening of polar extract

Phytochemical analysis of the polar extract of *G. wallichianum* was conducted following the methodology described in [36]. The presence of various compounds, including phenolics (ferric chloride test), coumarins (sodium hydroxide test), lignins (Labat test), saponins (foam test), flavonoids (lead acetate test), alkaloids (Wagner test), sterols (Salkowski test), leucoanthocyanins (isoamyl alcohol test), and glycosides (sodium hydroxide test), was evaluated. The biosynthesized AgNPs were characterized and evaluated for antibacterial and Hg^2+^ sensing applications.

### Synthesis of *G. wallichianum* mediated Ag NP*s*

A fresh *G. wallichianum* extract solution was prepared by dissolving 85 mg of the polar extract in 100 mL of DI water and mixing it with 5 mM AgNO_3_ (85 mg/100 mL DI water). The mixture was exposed to sunlight, and the color change was observed at 2, 5, 8, 10, and 15 min according to the method described in [25] with some adjustments. The synthesis was performed under direct sunlight (i.e., the entire spectrum from UV to IR, with a UV index between 7 and 10 as measured by a mobile app) on sunny days with clear skies between 10 a.m and 2 p.m. The use of sunlight in this process emphasizes the cost-effectiveness and sustainability of the green synthesis method. The AgNPs prepared using *G. wallichianum* were centrifuged at 13,000 rpm for 20 min at approximately 25 °C. The synthesized NP*s* were then dried at 45 °C for 24 h and labeled as GW@AgNPs for further use.

### Characterization

The synthesized AgNPs were characterized using various analytical and spectroscopic techniques. UV-Vis spectroscopic analysis was performed in the range of 200–700 nm with a UV-Vis spectrophotometer (UV-1700 Pharmspec, Shimadzu) to observe color changes in AgNPs from yellowish-brown to reddish-brown and finally to reddish-black as particle size increased at 2, 5, 8, 10, and 15 min. The FTIR spectra of the polar extract of *G. wallichianum* and GW@AgNPs were obtained using a Shimadzu FTIR 8400 S spectrometer in the range of 500–4000 cm^− 1^ with the KBr pellet procedure. The crystal structure of GW@AgNPs was investigated by PXRD using Jeol X-ray diffractometer system JDX-3532 using monochromatic X-rays (in the range of 20-80^o^, 2θ) to measure the intensity of reflected radiation. The average crystallite size was determined using the Debye-Scherrer equation (Eq. 1), and the lattice parameter was calculated using Eq. 2. SEM (JEOL JSM-5910) was performed to investigate the surface morphology of GW@AgNPs. Energy-dispersive X-ray (EDX) analysis (JEOL JSM-5910) was performed to qualitatively and quantitatively determine the elemental composition. The hydrodynamic dimensions of the nanocrystals and the surface charge of the GW@AgNPs were determined using a Malvern Zetasizer Nano ZS (ZEN3600).

1 $$ D = \frac{{k\lambda }}{{\beta \mathrm{Cos} \theta }} $$

Herein, “K” is the Scherrer constant, with a value of 0.9. The value of the X-ray source wavelength “λ” used in this study was 0.15406 nm. The full width at half maximum (FWHM) of the analyzed peaks in radians is represented by the symbol β. θ represents the orientation of the peak in radians.

For a face-centered cubic (fcc) crystal system, the relationship between the interplanar distance dhkl, and the lattice parameter a for planes with Miller indices (hkl) is given by the following equation (Eq. 2):

2 $$ {\mathrm{dhkl}} = \frac{{\mathrm{a}}}{{\sqrt {h^{2} + k^{2} + k^{2} } }} $$

### Antibacterial activity

To subculture the bacterial strains, MH agar (Oxoid), a medium for bacterial inoculum growth, was prepared by adding 4 g of agar to 100 mL of deionized (DI) water [37]. The pH of the solution was adjusted to 7.0 before autoclaving. Then, 25 mL of the solution was added to each Petri dish under the laminar flow cabinet. The Petri dishes were labeled, and a culture of *S. epidermidis* (ATCC 13883) was inoculated into one plate. The same procedure was repeated for *K. pneumoniae* (ATCC 14990) and *E. coli* (ATCC 11775). All Petri dishes were incubated at 37 °C for 24 h. The turbidity of the bacterial cultures was examined to confirm that the bacterial strains were viable and actively growing, which is essential for accurate testing of the antibacterial activity of synthesized GW@AgNPs.

Testing of the antibacterial activity of GW@AgNPs was performed according to the procedure described by Assad et al. (2025) and Roheen et al. (2025), with some amendments [37, 38]. MH agar (6.3 g) was completely dissolved in 120 mL of DI water. The Petri dishes and agar solution were autoclaved at 121 °C for 15 min. A 25 mL aliquot was poured into each sterilized Petri dish and allowed to set for 20 min. Bacterial strains were added to each Petri dish using the spread plate method. Wells were made in the Petri dishes and labeled alphabetically. Then, 30 µg/mL of erythromycin as a positive control, 30 µg/mL of GW@AgNPs, 30 µg/mL of plant extract, and DI water as a negative control were added to the labeled wells, and the plates were incubated at 37 °C for 24 h. The entire procedure was performed in triplicate in a sterilized laboratory environment.

### Determination of minimum inhibitory concentration and minimum bactericidal concentration

The microdilution technique, following an established protocol described in [40], was used to determine the minimum inhibitory concentration (MIC) and minimum bactericidal concentration (MBC). A 0.5 McFarland standard (1 × 10^8^ CFU/mL) was used to prepare suspensions of the tested bacterial strains. The standard broth microdilution method was used to determine the MIC of GW@AgNPs [41]. Various concentrations, ranging from 5 to 40 µg/mL, were used to determine the MIC. Positive control tests with standard bacterial strains and broth, as well as negative control tests with 40 µg/mL AgNPs, were also performed. The samples were then incubated at 37 °C for 24 h. After MIC determination, 5 µL from all tubes showing no visible bacterial growth was cultured on MH agar and incubated at 37 °C for 24 h. After incubation, the MBC values the minimum concentration required to inhibit bacterial growth by killing the bacteria were calculated.

### Colorimetric sensing of heavy metal ions using GW@AgNPs

To evaluate the efficiency of green synthesized GW@AgNPs for the selective detection of heavy metal ions in an aqueous medium, solutions of metal ions (Pb^2+^, Cd^2+^, Ni^2+^, Co^2+^, and Hg^2+^) were added to the GW@AgNP suspension following the protocol of [42]. This experiment was conducted in separate test tubes, each containing 1 mL of GW@AgNP suspension and 1 mL of a specific metal ion solution. The UV-Vis spectra of the mixtures were recorded. A discernible change in the UV-Vis spectrum or the color of the GW@AgNP solution after adding metal ions can serve as the basis for the selective detection of a particular metal ion.

A major color change was noticed in the Hg^2+^ containing solution. Moreover, a calibration curve was established by recording the spectra of various concentrations of Hg^2+^ between 1 and 100 µM. This was performed to determine the lowest level of Hg^2+^ that could be detected using GW@AgNPs. To determine any interference in the detection of Hg^2+^, 1 mL of GW@AgNPs was combined with different metal ions, Pb^2+^, Cd^2+^, Ni^2+^, and Co^2+^, in the presence of Hg^2+^. To analyze Hg^2+^ in real water samples, the percentage recovery, change in absorption (ΔA), and limit of detection (LOD) were determined using the following equation (Eq. 3). The limit of detection (LOD) was calculated following the IUPAC guidelines [43].

3 $$ LOD = \frac{{3 \times \sigma }}{S} $$

where σ is the standard deviation of the response (noise), and S is the slope of the calibration curve. A factor of three ensured a confidence level of approximately 99%, indicating that the signal could be reliably distinguished from the background noise.

The change in absorption was calculated using the following equation (Eq. 4):

4 $$ \Delta A = \left( A \right)final - \left( A \right)initial $$

where (A) final is the absorbance after the addition of the analyte, and (A) initial is the absorbance of the blank or the initial sample.

The percentage recovery was calculated using the formula (Eq. 5):

5 $$ \% ~Recovery = \frac{{Found~\;Concentration}}{{Known~\;Concentration}} \times 100 $$

where the “Found Concentration” is the measured concentration of Hg^2+^ in the spiked sample, and the “Known Concentration” is the spiked concentration of Hg^2+^. The experiment was conducted triplicate.

### Statistical analysis

The results are expressed as mean ± standard deviation. Statistical analyses were performed using Origin Pro 2021 and Microsoft Excel 2016. One-way analysis of variance (ANOVA) was performed to determine the significance of differences in antibacterial activity (zone of inhibition), MIC, and MBC values among the different treatments. Statistical significance was set at *p* < 0.05.

## Results and discussion

### Phytochemical analysis

The findings of the phytochemical analysis of the polar extract of *G. wallichianum* are presented in Table 1S. Quantitative analyses of the phytochemical components that reduced and capped the AgNPs were also conducted. Analysis of the polar extract of *G. wallichianum* revealed that it is a good source of phytochemicals, including phenols, alkaloids, flavonoids, glycosides and saponins. The findings of this study are consistent with the findings of previous publications [44].

### Green synthesis of GW@AgNPs

**Fig. 1:**
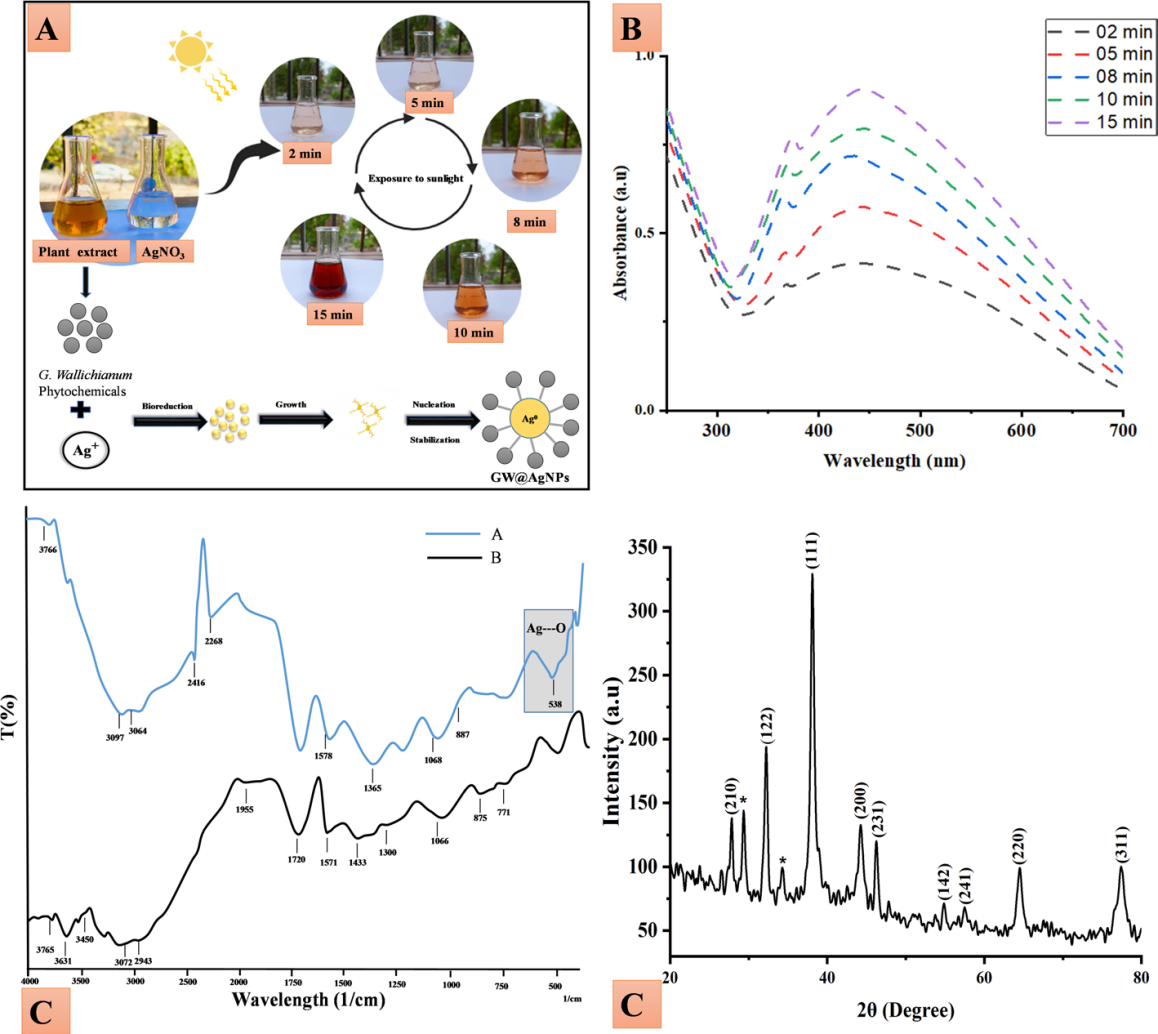
**A** Schematic representation of the green synthesis of GW@AgNPs, showing a color change under sunlight at 2, 5, 8, 10, and 15 min, **B** UV-vis spectra of AgNPs prepared with *G. wallichianum* (5 mM) at different reaction times, **C, A** FTIR spectra of AgNPs produced by polar extract of *G. wallichianum* (5 mM) after exposure to diffuse sunlight for 15 min, (B) spectra of the pure polar extract of *G. wallichianum* and **D** PXRD spectra of AgNPs synthesized with the polar extract of *G. wallichianum*

When a mixture of AgNO₃ and *G. wallichianum* extract is exposed to sunlight, hydrated electrons are produced in the system. These electrons reduce monovalent silver cations to zerovalent silver atoms [45]. The resulting zero-valent silver atoms are generally in the nanometer size range. UV irradiation under sunlight and phytochemicals in plant extracts can be used as a green method for synthesizing AgNPs. The color changed from yellowish-brown to reddish-brown, indicating the green synthesis of AgNPs, as illustrated in Fig. 1A.

The reduction of Ag^+^ to Ag^0^ during the green synthesis of GW@AgNPs could be attributed to various phytochemicals present in the polar extract of *G. wallichianum*, such as phenolics, flavonoids, alkaloids, saponins and glycosides (as shown in Sect.  3.1). These molecules are generally considered electron donors in redox reactions due to functional groups including hydroxyl (-OH), amine (-NH₂), and carbonyl (C = O). For instance, the tautomeric transformation of flavonoids and phenolics makes reactive hydrogen atoms available to reduce Ag^+^ to Ag^0^, and their oxidized forms can also serve as stabilizers or capping agents for the NPs. This mechanism is also supported by the observed FTIR shifts (Sect.  3.4), which show significant changes in the -OH, C = O and N-H stretching regions, indicating their involvement in the reduction and stabilization reactions. This dual role is consistent with previous reports where natural plant extracts acted in both the nucleation and growth of metallic NPs [46]. This approach also helps avoid the use of toxic and hazardous reducing agents in the environment [30]. In the present study, a polar extract of *G. wallichianum* was used as a green reducing agent for the synthesis of AgNPs under sunlight. The synthesis was carried out using a 5 mM AgNO_3_ solution, followed by mixing the reactants to form an Ag–[*G. wallichianum*] complex, which exhibited a yellowish-brown to reddish-brown color.

### UV-visible spectroscopic analysis

The synthesis of AgNPs using the polar extract of *G. wallichianum* was investigated using UV-Vis spectrophotometry in the range of 700 –200 nm. The solution color changed over time as Ag^+^ was reduced to Ag^0^ in sunlight. The collective oscillations of conduction electrons in the AgNPs played an important role in the strong absorption associated with the Surface Plasmon Resonance (SPR) phenomenon in the visible region for the 5 mM AgNO_3_ solution, showing the synthesis of AgNPs at the corresponding reaction times of 2, 5, 8, 10, and 15 min [25]. The wavelength of the absorbed light (bathochromic shift) increased with the size of the AgNPs over time [47]. The UV-Vis spectroscopic results are consistent with the previously documented synthesis of AgNPs using plant extracts and sunlight [48]. Furthermore, the absorption intensity increased from 2 to 15 min, indicating a uniform increase. The SPR transition exhibited a color shift from yellowish-brown to reddish-brown, consistent with the changes in the size and response time of the AgNPs [49]. The color change to reddish-brown is associated with SPR. The usual SPR peaks in the UV-Vis spectra of AgNPs mediated by *G. wallichianum* appeared at 438 nm, 439 nm, 441 nm, 443 nm, and 445 nm, as Ag⁺ ions were reduced by *G. wallichianum* extract, as illustrated in Fig. 1B. Our results are in good agreement with previously published findings on the green synthesis of AgNPs using plant extract [50]. Several reports have stated similar UV-Vis spectroscopy using plant extracts, such as *Peganum harmala* L [51]., *Capparis nummularia* DC [30]., *Oroxylum indicum* (L.) Kurz [52], and *Breynia nivosa* Forst. & G.Forst [53].

### FTIR spectroscopy

FTIR spectroscopy was performed to confirm the functional groups present in the polar extract of *G. wallichianum* and their interactions with the green-synthesized GW@AgNPs. An excitation at 3308–3852 cm^− 1^ was detected in the FTIR spectrum of the polar extract of *G. wallichianum*, indicating the presence of N-H and OH-containing groups. The OH-containing excitation was reduced in the spectra of GW@AgNPs, indicating their interaction with the green-synthesized GW@AgNPs [54]. The peaks in the range (2841–2983 cm^− 1^) correspond to the symmetric and asymmetric stretching of C-H bonds in aliphatic hydrocarbons [55]. The presence of these peaks in both GW and GW@AgNPs suggests that the aliphatic chains of the plant extract remained intact and may have contributed to the hydrophobic capping of the NP*s*, which helped stabilize them. Peaks around 2100–2260 cm^− 1^ are typically associated with C ≡ C/C ≡ N stretching vibrations found in alkynes. This peak in the GW extract may have originated from phytochemicals containing unsaturated alkynes. However, in GW@AgNPs, the shift or reduction in intensity suggests the possible involvement of alkynes in the interaction or stabilization of AgNPs [54, 55]. In GW@AgNPs, the shift of the peak at 1737 cm/1 suggests that carbonyl groups are actively involved in binding to the nanoparticle surface and serve as stabilizing agents through coordination with silver ions. The FTIR spectra showed that the polar extract of *G. wallichianum* shifted the alkyl amine stretching (1199 cm^− 1^ 1272 cm^− 1^) and C-H stretching (824 cm^− 1^ 979 cm^− 1^) to form a large band for GW@AgNPs with the polar extract of *G. wallichianum* (5 mM), as shown in Fig. 1C (A and B). FTIR analysis showed that GW@AgNPs had distinct bands at 605 cm^− 1^, indicating the interaction of AgNPs with oxygen groups in the plant extract. This interaction refers to the interaction between AgNPs and OH-containing groups [57].

The presence of hydroxyl (–OH) and carbonyl (C = O) groups from plant phytochemicals has been repeatedly reported as participating in the reduction and stabilization of Ag^+^ to zero-valent silver in studies utilizing the green synthesis approach. Several studies have reported that the FTIR spectra of plant extracts show broad O-H stretching bands (3300–3500 cm^−1^) and C = O or related carbonyl/amide peaks (1650–1750 cm^−1^) which shift in frequency or intensity upon NPs formation, suggesting their role as electron donors, and as capping or stabilizing agents [57, 58]. For example, in a study using aqueous leaf extract of *Micrargeria wightii*, FTIR analysis confirmed that plant metabolites bearing –OH, C = O, and amide (-CO-NH_2_) groups mediated the reduction of Ag^+^ to Ag^0^ [60]. Similarly, in the biosynthesis of AgNPs via plant extracts, characteristic SPR peaks in UV-Vis spectra (around 430–445 nm) coincided with FTIR-confirmed presence of phenolic, carbonyl, and other functional groups, illustrating that these moieties act as both reducing agents and stabilizers [61]. In the present study, the FTIR spectra following AgNP formation support the finding that these groups were consumed or rearranged during reduction and subsequently served as capping ligands to stabilize the nanoparticles. The FTIR spectra of GW@AgNPs and the polar extract of *G. wallichianum* exhibited prominent peaks, as shown in Table 1.

**Table 1:** FTIR analysis of green synthesized AgNPs using polar extract of *G. wallichianum*

Wavenumber (cm^−1^)	Functional group	References
3113–3853	N–H and O–H stretching	[62]
2841–2943	C–H stretching	[63]
2202–2235	C≡C / C≡N stretching	[64]
1559–1737	C=O and N–H bending	[65]
1199–1272	C–N stretching	[66]
824–972	C=O stretching	[67]
605	Ag–O interaction	[10]

### PXRD studies

The PXRD study confirmed the crystal phase of AgNPs produced with *G. wallichianum*. Figure 1D shows the typical PXRD pattern of the AgNPs prepared using *G. wallichianum.* The AgNPs exhibited distinct diffraction peaks at 2θ. Bragg reflection peaks appeared at 2θ values of 27.81°, 32.16°, 38.12°, 44.3°, 46.21°, 54.83°, 57.39°, 64.42°, and 77.45°, corresponding to the (210), (122), (111), (200), (231), (142), (241), (220), and (311) planes of pure silver based on the face-centered cubic (fcc) structure (JCPDS, File No. 04-0783). These results are consistent with those of previous studies [67, 68]. According to the literature [49], the intensity of the AgNPs indicates a high degree of crystallinity in the AgNPs. The diffraction peaks confirmed the face-centered cubic geometry of Ag. Additional signals were observed owing to the presence of plant extracts in the sample. Plant extracts contains various bioactive compounds, including phenols, flavonoids, alkaloids, and other secondary metabolites. These compounds participate in both the reduction and stabilization of silver ions to AgNPs. Their interaction with AgNPs produces additional signals corresponding to the organic compounds [70].

Using the Debye-Scherrer equation (Eq. 1), the average crystallite size was 14.88 nm. The calculated lattice parameter (Eq. 2), *a* for the fcc reflections is approximately 5.16 Å. This value was averaged from the lattice parameters calculated using valid fcc Miller indices (Table 2).

**Table 2:** Lattice parameters of the GW@AgNPs face-centered cubic (fcc) structure

No	Peak position 2θ (degree)	Interplaner spacing D (Å)	Miller indices (hkl)	Lattice (Å)
1	27.81	3.20	210	
2	32.16	2.78	122	
3	38.12	2.35	111	
4	44.3	2.04	200	
5	46.21	1.96	231	5.16
6	54.83	1.67	142	
7	57.39	1.60	241	
8	64.42	1.44	220	
9	77.45	1.23	311	

### SEM - EDX analysis

**Fig. 2:**
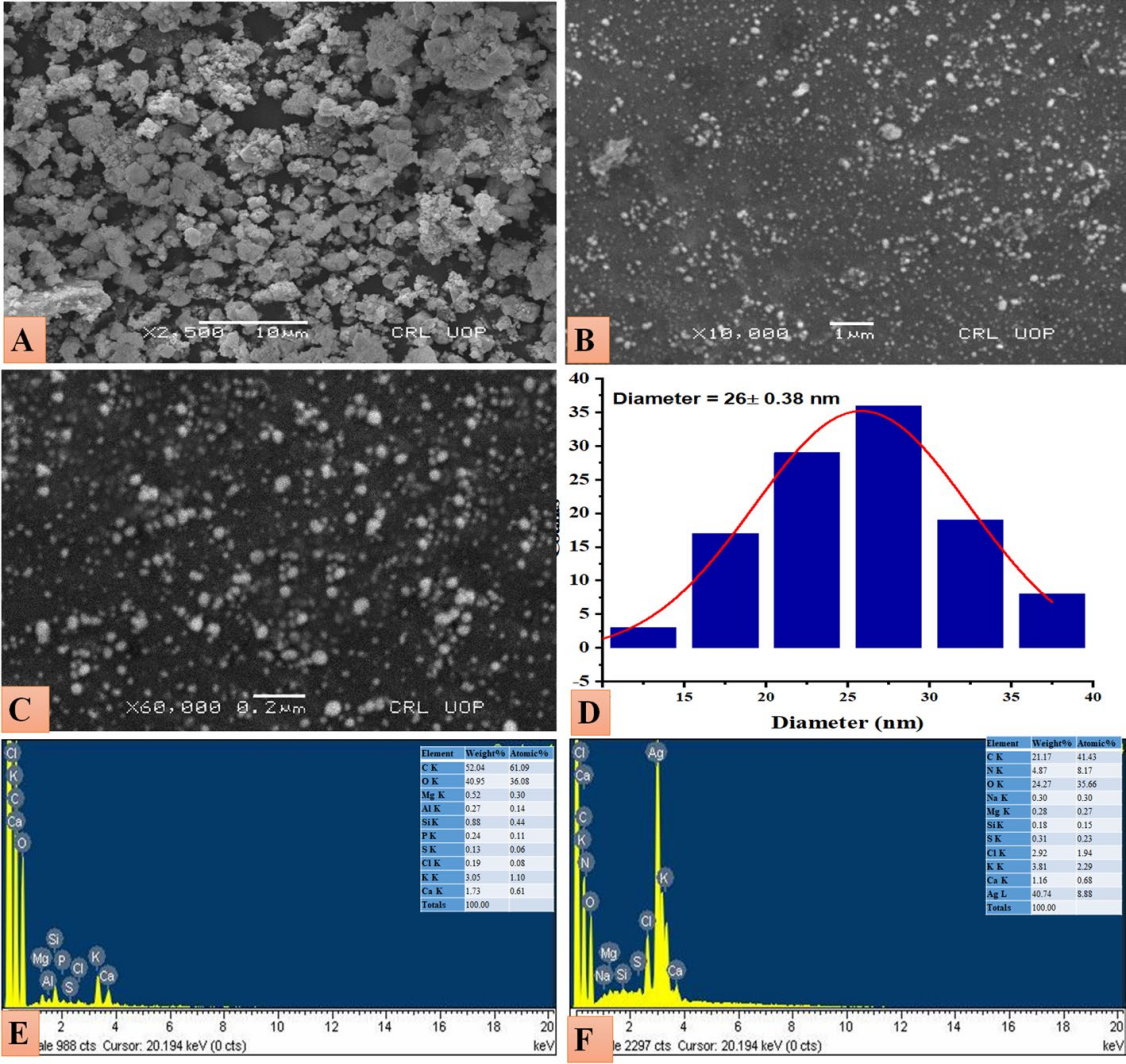
SEM-EDX analysis of green-synthesized GW@Ag. **A–C** SEM images at different magnifications (10,000× to 50,000×) showing nearly spherical nanoparticles with visible aggregation; all images include scale bars (500 nm and 1 μm), **D** Histogram of average particle size, **E** EDX spectra of the plant extract, **F** EDX spectra of GW@AgNPs

SEM-EDX was performed to verify the surface morphology, average size, and chemical composition of GW@AgNPs [71]. The morphology of GW@AgNPs showed nearly spherical structure, as shown in Fig. 2A, B, C. The average size of GW@AgNPs was 25.83 ± 0.38 nm, as shown in the histogram in Fig. 2D. According to Devatha et al. (2016), the OH groups in plant extracts are the primary factors leading to variations in NP size [72]. Plant extracts with high concentrations of reductive biomolecules can accelerate AgNP synthesis [72, 73]. The smaller particle size may be attributed to the GW extract, which acted as a capping and stabilizing agent [75]. A few large GW@AgNPs aggregates, observed in the SEM analysis, were formed as a consequence of the aggregation of smaller NPs. SEM-EDX was used to detect the presence of Ag. Figure 2E shows the EDX spectrum of the polar extract of *G. wallichianum*. The AgNPs obtained from the polar extract of *G. wallichianum* were detected as a distinct SPR absorption band in the EDX spectra. GW@AgNPs exhibited a substantial absorption peak at 3 keV, which was formed by SPR [76]. The signal band (40.74 weight%) in Fig. 2F shows Ag along with other elements. The other elements are attributed to the GW@AgNPs capped with the plant extract, which were also found in the EDX spectra of the polar extract of *G. wallichianum* [77].

### DLS analysis

**Fig. 3:**
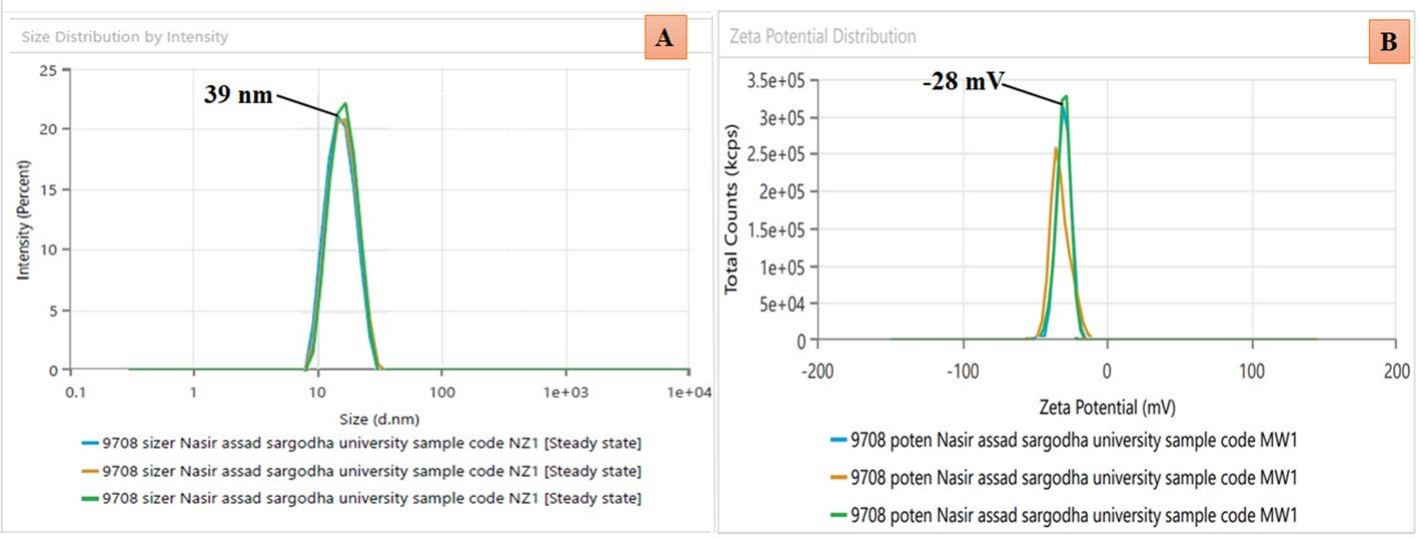
Dynamic light scattering measurement of green synthesized GW@AgNPs **A** particle size analysis, **B** zeta potential analysis

The hydrodynamic size of the green-synthesized AgNPs was investigated using DLS analysis in a colloidal solution. DLS is one of the most commonly used methods for determining the size characteristics of nanoparticles (NPs). The average size of the AgNPs was determined to be 39 ± 3.7 nm, with a Polydispersity Index (PDI) of 0.293 (Fig. 3A). This size is relatively larger than that observed in SEM analysis (~ 25 nm), which is expected due to the inherent differences between the two techniques. SEM measures the size of the dry core of nanoparticles under high vacuum, and DLS evaluates the particles in their hydrated, colloidal state, including the metallic core, phytochemical capping agents, and solvation layer. This leads to a larger hydrodynamic diameter in the DLS. The size difference confirmed the successful surface functionalization and good colloidal stability of the AgNPs in an aqueous medium [78].

The zeta potential study indicated the stability of GW@AgNPs. The voltage quantified from the charge developed on the surface of the GW@AgNPs was − 28 ± 2.4 mV, demonstrating a good degree of stability (Fig. 3B). The presence of negative charges on the NPs leads to electrostatic repulsion, which contributes to the stabilization of the NPs in a colloidal solution. In addition, a strong negative charge increases the stability. Negatively charged NPs prevent aggregation and help control their shape and size [79]. The results of our study are in good agreement with those of previous studies [77, 79].

### Thermogravimetric and differential scanning calorimetry analysis

Thermogravimetric analysis (TGA) and differential scanning calorimetry (DSC) were used to evaluate the thermal behaviors of the synthesized nanoparticles. The TGA curve exhibited minimal weight loss below 200 °C, indicating the absence of moisture and volatile organic components. A major thermal event was observed at approximately 653 °C, corresponding to the thermal behavior of metallic silver. Remarkably, approximately 70.47% of the original sample weight remained at 818 °C, which is consistent with the high thermal stability and inertness of AgNPs as shown in Fig. 1S. In contrast, silver oxide (AgO) typically decomposes between 200 and 300 °C, releasing oxygen and converting to metallic silver. The absence of such decomposition in the current profile confirms the reduction of Ag⁺ to metallic Ag during the green synthesis. Thus, the TGA/DSC results strongly support the successful formation of stable AgNPs rather than AgO.

### Antibacterial activity

Antibiotic resistance has become a major problem associated with microbial diseases in recent years. According to the World Health Organization (WHO), this contributes to the increasing difficulty in treating infections, such as foodborne diseases, blood poisoning, and pneumonia [81]. Since their introduction in the 20th century, bacteria have developed defense mechanisms that weaken the effectiveness of antibiotics or render them ineffective [82]. Many complex mechanisms, including increased expression of efflux pumps that remove drugs from bacterial cells, genes that alter the binding substrate and thus the drug targets, and proteins and enzymes that modify the drug [83], are involved in drug resistance. Currently, multidrug-resistant bacteria (MDR) pose the greatest risk to human health and healthcare systems [83, 84]. Therefore, new ways to treat bacterial infections need to be found; nanomaterials have shown promise as potential antibacterial agents [86, 87].

**Fig. 4:**
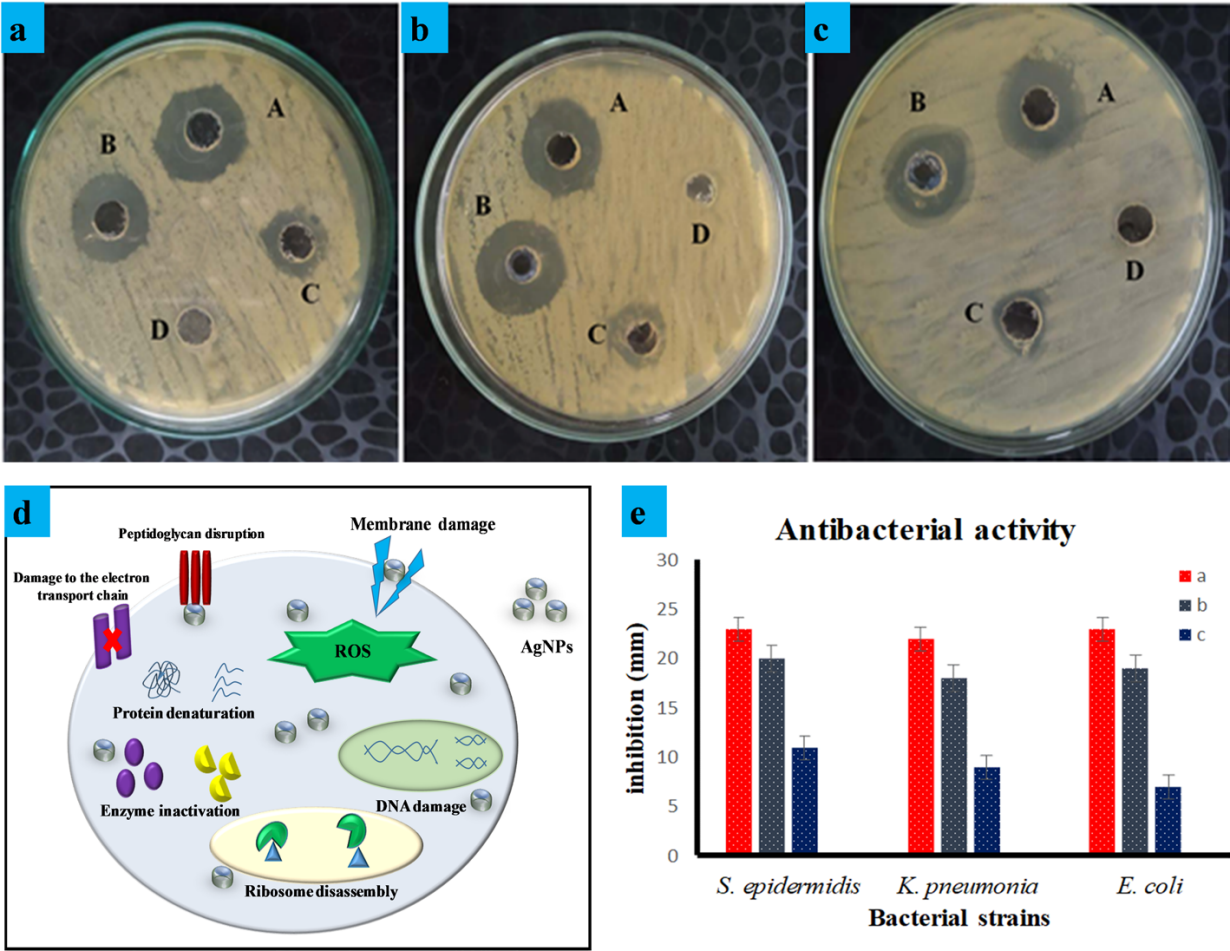
Zones of inhibition of *G. wallichianum-mediated* nanoparticle *against* **a** *S. epidermidis (ATCC 13883)*, **b** *K. pneumoniae (ATCC 14990)*, and **c** *E. coli* (*ATCC 11775*). Where (A) is the positive control (erythromycin), (B) Ag NP*s*, (C) plant extract, (D) negative control (DI water), and **d** proposed mechanism of antibacterial action of GW@AgNPs and **e** Antibacterial activity of *G. wallichianum* mediated nanoparticles (a) positive control (b) AgNPs (c) plant extract

The AgNPs mediated by *G. wallichianum* were tested against various pathogenic bacteria, including *S. epidermidis*, *K. pneumoniae*, and *E. coli*, using the well diffusion method. The synthesized GW@AgNPs exhibited antibacterial activity against both Gram-negative and Gram-positive bacterial strains. The zones of inhibition (ZOI) of AgNPs against *S. epidermidis*, *K. pneumoniae*, and *E. coli* were 20, 18, and 19 mm, respectively. Our results are comparable to those of the standard antibiotic erythromycin and greater than those of the plant extract. Figure 4a–c shows the diameter of the ZOI (mm) with plant extract and AgNPs solution around each well, and Fig. 4e shows a graphical representation of the ZOI. Therefore, the AgNPs prepared with *G. wallichianum* have shown good antimicrobial activity against *S. epidermidis*,* K. pneumoniae*, and *E. coli.* These results are consistent with those of a previous study [88]. The polar extract of *G.*

*wallichianum* exhibited antimicrobial activity against *S. epidermidis*, *K. pneumoniae*, and *E. coli.* Consistent with earlier findings, the present data demonstrate that, compared to Gram-positive bacteria (*S. epidermidis*), Gram-negative bacteria (*K. pneumoniae* and *E. coli*) exhibit higher resistance to AgNPs [89]. *K. pneumoniae* and *E. coli* may be less vulnerable to AgNPs due to differences in their cell wall composition or due to the fact that lipopolysaccharides capture and block the charges of AgNPs [10]. One-way ANOVA confirmed statistically significant differences (*p* < 0.05) in the inhibition zone values among AgNPs, plant extract, and control for all tested bacterial strains (*S. epidermidis*, *K. pneumoniae*, and *E. coli*), validating the superior antibacterial efficacy of the green-synthesized AgNPs (Table 2S).

According to literature reports, the antibacterial activity of AgNPs is commonly attributed to mechanisms involving ROS generation, membrane disruption, and biomolecular damage, including protein denaturation and deoxyribonucleic acid (DNA) fragmentation [90]. Le Ouay and Stellacci (2015) provided a comprehensive explanation of the mechanisms by which AgNPs interact with bacteria [91]. Their study described the role of Ag^+^ species, which are the main cause of antibacterial activity. Ag^+^ is highly attracted to amines, phosphates, and thiols, which are common in biological systems. It can also react with peptides and DNA [92]. Its weak selectivity is assumed to enable Ag^+^ to react with multiple targets simultaneously, thereby promoting bacterial death [93]. The oxidative dissolution of AgNPs disrupts the bacterial membrane by creating Ag^+^ ions. The size of the nanoparticles directly impacts the antibacterial activity, as smaller NPs dissolve faster and release more Ag^+^ ions. The major mode of action of AgNPs as antibacterial agents is the generation of reactive oxygen species (ROS), which cause oxidative stress and cell death [94]. Two processes regulate the increase in ROS levels induced by the AgNPs. The first is based on the presence of reactive groups, such as radicals or oxidants, usually found on the surface of nanoparticles, which directly participate in ROS formation [84, 93]. The second mechanism involves the inhibition of protective responses against ROS, such as the destruction of glutathione (a normal antioxidant within cells), which reduces the cell’s ability to counteract ROS formation [97]. The connection between ROS formation and nanoparticle properties, including size and shape, is clear, as is the Ag^+^ associated process [98]. Although the actual mechanisms of toxicity are not yet fully understood, a correlation exists between surface features and toxicity pathways. The antibacterial mechanism of GW@AgNPs is attributed to ROS generation, membrane disruption, and biomolecular damage, including protein denaturation and DNA fragmentation (Fig. 4d). Future studies are required to perform protein leakage and ROS quantification assays to validate these findings.

### MIC and MBC of GW@AgNPs

The MIC*s* and MBC*s* of GW@AgNPs against the three bacterial species were estimated using the broth dilution method. The MIC was calculated by co-culturing bacteria for 24 h with different concentrations of 40–5 µg/mL GW@AgNPs. The MIC is defined as the lowest concentration that inhibits bacterial growth. GW@AgNPs had MICs of 15 and 25 µg/mL (1 mg/mL) against *S. epidermidis* and *K. pneumoniae* and 25 µg/mL (1 mg/mL) against *E. coli* (Table 3). One-way ANOVA of the MIC values confirmed statistically significant differences (*p* < 0.05) between the treatments with AgNPs and plant extracts for all bacterial strains tested, emphasizing the superior antibacterial potency of AgNPs (Table 3S). The MBC values for *S. epidermidis*, *K. pneumoniae*, and *E. coli* were 20, 25, and 30 µg/mL (1 mg/mL), respectively, as shown in Table 3. One-way ANOVA of the MBC values also showed statistically significant differences (*p* < 0.05) between the treatments with AgNPs and plant extracts, supporting the enhanced bactericidal activity of AgNPs (Table 4S). Gram-positive bacteria showed greater susceptibility to GW@AgNPs compared to Gram-negative bacteria, as shown by the results of the antimicrobial susceptibility test. The size and zeta potential of AgNPs are important factors in their ability to penetrate the cell membrane and effectively kill bacteria [99]. More specifically, the antibacterial activity of smaller GW@AgNPs is enhanced because of their smaller size and larger surface area compared with larger particles [100]. Moreover, the spherical shape of AgNPs is crucial for their absorption, which leads to the death of the microbes [101].

**Table 3:** MIC and MBC of GW@AgNPs (+ turbidity, − clarity)

Bacterial strain	GW@AgNPs (µg/mL)								MIC (GW@AgNPs) (µg/mL)	MBC (GW@AgNPs) (µg/mL)
	5	10	15	20	25	30	35	40		
*K.* *pneumoniae*	+	+	+	+	−	−	−	−	25 ± 0.4	25 ± 0.5
*S. epidermidis*	+	+	+	+	−	−	−	−	15 ± 0.4	20 ± 0.4
*E. coli*	+	+	−	−	−	−	−	−	25 ± 0.3	30 ± 0.2

Results are specified as mean ± standard error and performed in triplicate

### Selective colorimetric detection of Hg^2+^

**Fig. 5:**
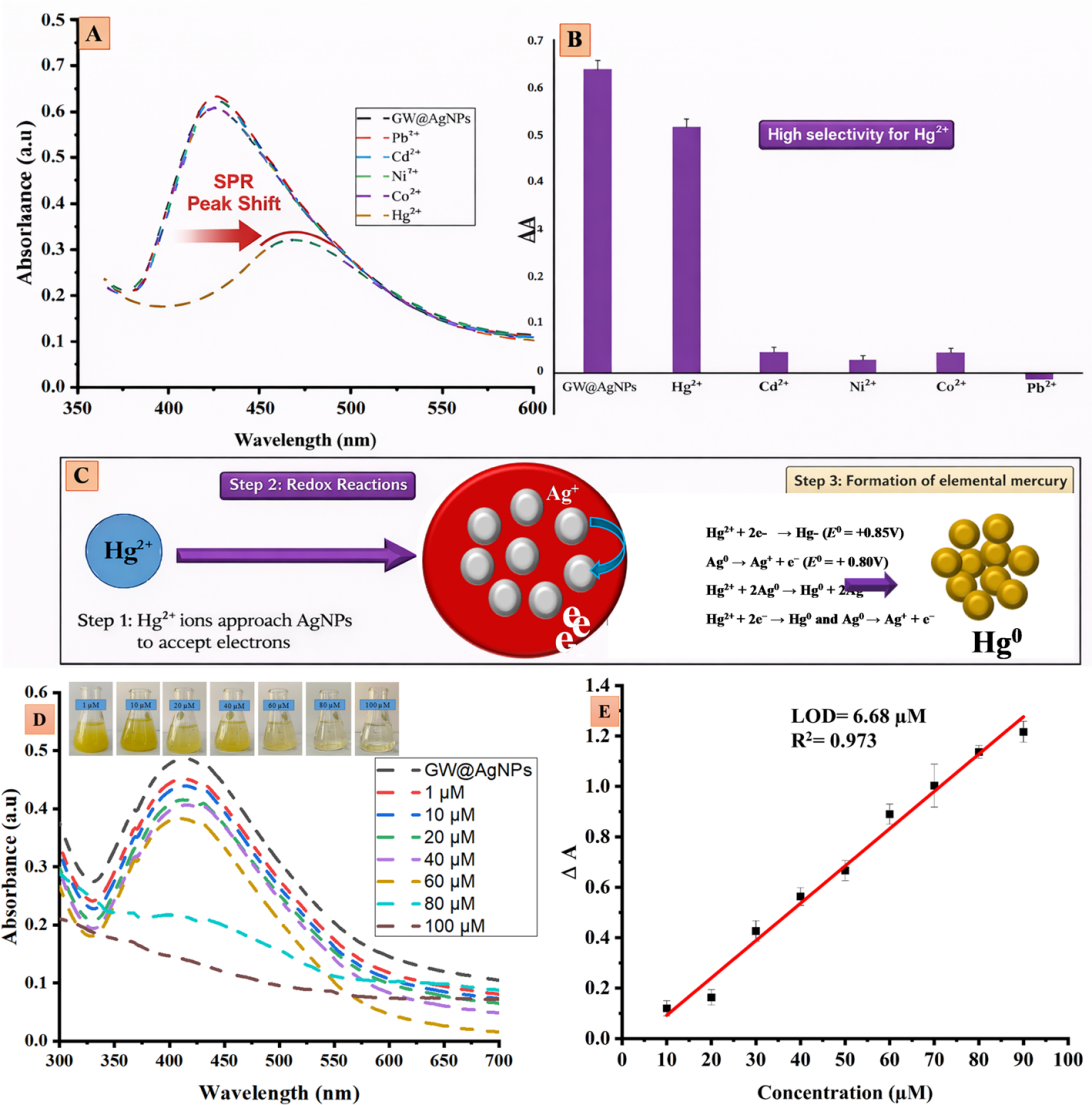
Colorimetric detection, **A** UV-Vis spectra of Pb^2+^, Cd^2+^, Ni^2+^, Co^2+^, and Hg^2+^, **B** corresponding changes in the UV-vis spectra of different metals and **C** redox mechanism of Hg detection using AgNPs, **D** UV-Vis spectra of Hg^2+^ at different concentrations, **E** calibration curve plotting the change in absorbance against the concentration of Hg^2+^

Recent advances have reinforced the potential of plant-mediated- green synthesis for producing functional AgNPs with dual roles in antimicrobial and heavy metal- detection applications [99, 100]. The sensory potential of GW@AgNPs was tested with 1 mM solutions of Pb^2+^, Cd^2+^, Ni^2+^, Co^2+^, and Hg^2+^ ions, and UV-Vis spectra of the suspensions were obtained. The addition of Pb^2+^, Cd^2+^, Ni^2+^, and Co^2+^ solutions did not change the color or UV-Vis spectra of the GW@AgNP suspensions, except for Hg^2+^, as shown in Fig. 5A, B. However, the Hg^2+^ solution caused a conspicuous and abrupt color change from yellowish-brown to colorless when added to the GW@AgNPs suspension. A UV-Vis spectrophotometer was used to quantify the color change. The UV-Vis spectra in Fig. 5A exhibit a clear decay and disappearance of the LSPR (Localized Surface Plasmon Resonance) peak of the GW@AgNPs suspension at approximately 420 nm, only after the addition of the Hg^2+^ solution. The redox chemistry of Hg^2+^ and GW@AgNPs showed a specific ability to recognize heavy metal ions in aqueous solutions. The colorimetric detection of Hg^2+^ ions using AgNPs is primarily driven by a redox reaction based on the standard electrode potentials. The standard reduction potential of the Hg^2+^/Hg⁰ couple (+ 0.85 V) is higher than that of the Ag⁺/Ag⁰ couple (+ 0.80 V), which enables spontaneous electron transfer from silver atoms (Ag⁰) in the NPs to Hg^2+^ ions.

This flow of electrons oxidizes Ag^0^ to Ag^+^ and reduces Hg^2+^ to elemental mercury (Hg^0^), interfering with the surface plasmon resonance (SPR) of the AgNPs (Fig. 5C) and inducing a color change [101, 102]. The redox potential of Hg ^2+^ enables electron transfer between AgNPs and Hg ^2+^ ions, whereas other ions such as Pb^2+^, Cd^2+^, Ni^2+^, and Co^2+^ do not show comparable redox behavior. These ions may weakly interact with the AgNP surface but do not produce the same pronounced color change because their redox potentials are not as compatible with Ag^0^ /Ag^+^ as that of Hg^2+^. This leads to high selectivity for Hg^2+^ detection, making AgNPs effective sensors for Hg^2+^ in the environment.

The redox reaction can be explained by the following half-reactions (Eqs. 6 and 7):

6 $$ Ag^{ 0} ~ \to ~Ag^{ + } ~ + ~e^{ - } \;\left( {E^{ \circ } ~ = ~ + 0.80~V} \right) $$

7 $$ Hg^{{2 + }} + 2e^{ - } \to Hg^{0} \;(E^{^\circ } = + 0.85V) $$

The overall redox reaction is given by Eq. (8).

8 $$ 2Ag^{0} + ~Hg^{{2 + }} \to ~2Ag^{ + } + ~Hg^{0} $$

This interaction caused a significant change in the optical characteristics of the resulting GW@AgNP suspension.

Figure 5D, E shows the results of the study on the optical characteristics of the GW@AgNP solution after adding varying amounts of Hg^2+^ ions (1-100 µM) to establish a calibration curve. Naked-eye observations indicated a fading of the yellowish color with increasing Hg^2+^ concentration, as shown in Fig. 5D. Simultaneously, the UV-Vis spectra recorded at these concentrations showed a clear blue shift and a sharp decrease in the intensity of the LSPR band at approximately 420 nm (Fig. 5D). The observed blue shift is explained by the Mie phenomenon, which states that the LSPR wavelength decreases and the Mie resonance frequency increases when the size of the GW@AgNPs changes. Compared to the control experiment (0 µM, black line), a significant decrease in absorbance was observed. To determine the LOD, a calibration curve was created by plotting the change in absorbance against the concentration of Hg^2+^ (Fig. 5E). The slope of this linear regression resulted in an LOD of 6.68 µM. The calculated LOD of 6.68 µM (~ 1.35 ppm) exceeds the WHO limit for drinking water (2 ppb); however, the sensor is highly selective and effective for detecting elevated Hg^2+^ levels in industrial effluents and contaminated environmental samples.

The LOD of Hg^2+^ achieved in this work (6.68 µM) is considered moderate compared to many recent nanosensors. For instance, a dual‑mode biosensing design demonstrated a colorimetric LOD of 36.3nM and a fluorescence LOD of 0.59nM [106]. Functionalized AgNPs sensors have achieved LODs as low as ~ 0.35 µM [107]. Although the current GW@AgNP‑based sensor demonstrates a relatively high LOD, it offers several advantages: green synthesis routes that require neither toxic reagents nor sophisticated instruments; rapid visible detection, enabling in situ analysis for field‑level screening and preliminary testing when Hg^2+^ concentrations are higher (e.g., industrial wastewater, contaminated effluents). For applications requiring trace‑level (nM) sensitivity, such as drinking water or ecological monitoring, additional optimization (e.g., surface functionalization, signal amplification, dual‑mode readout) would be required.

### Interference study of GW@AgNPs

**Fig. 6:**
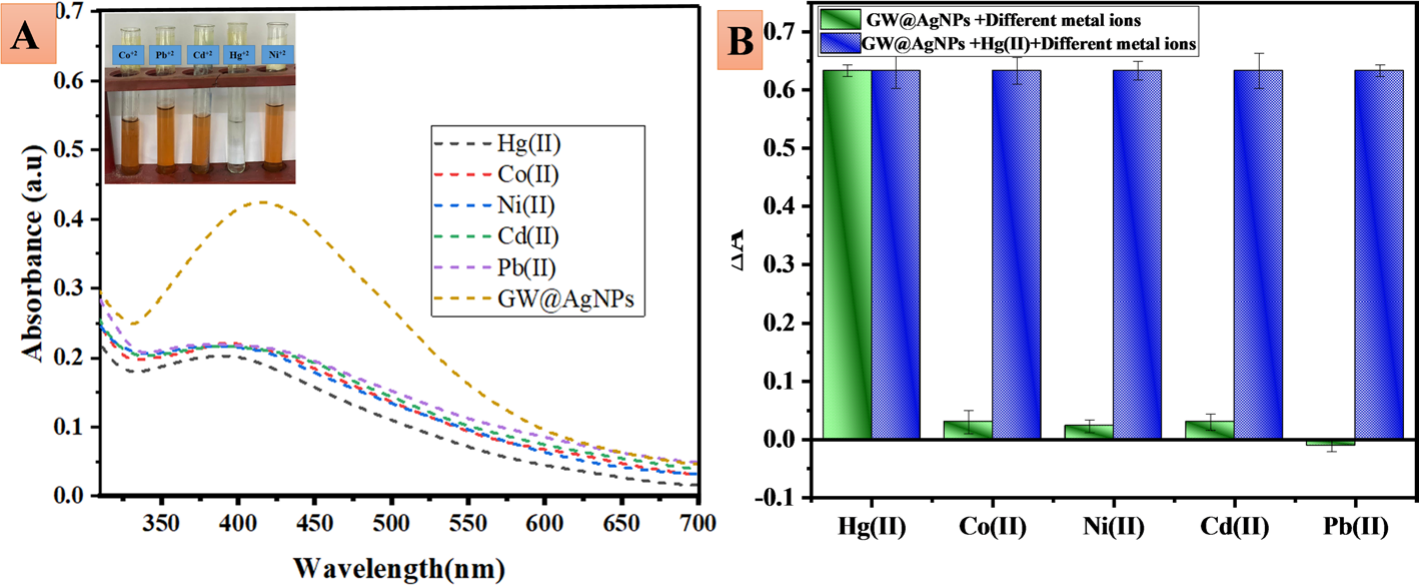
**A** UV-Vis spectra of 5 mM, GW@AgNPs + 100 µM Hg^2+^ after addition of 1 mM solution of Pb^2+^, Cd^2+^, Ni^2+^, and Co^2+^, **B** bar graph representing the results of interference of the different metals

A study was carried out to investigate the potential interference caused by different metal ions in the detection of Hg^2+^. The experiment involved analyzing the UV-Vis spectra of a 5 mM GW@AgNPs solution (1 mL) and 1 mL Hg^2+^ (100 µM), in the presence of 1 mL of interfering metal ions; Pb^2+^, Cd^2+^, Ni^2+,^ and Co^2+^ salts (1 mM) as shown in Fig. 6A. These results show that there was no chemical reaction between GW@AgNPs and the other metal ions present in the salt solution. However, GW@AgNPs showed a particular interaction with Hg^2+^ (Fig. 6A). The change in absorbance (ΔA) of GW@AgNPs is shown in Fig. 6B. The green bars indicate the differences in the absorbance of 5 mM GW@AgNPs (1 mL) + 1 mL solution for each of the following metal ions: Pb^2+^, Cd^2+^, Ni^2+,^ and Co^2+^. The blue bars represent the change in the absorbance of 5 mM GW@AgNPs + 100 µM Hg^2+^ when exposed to a 1 mM solution of Pb^2+^, Cd^2+^, Ni^2+,^ and Co^2+^. This indicates that GW@AgNPs exhibit considerable selectivity for Hg2 + detection.

### Elemental mapping and EDX analysis of AgNPs for mercury detection

**Fig. 7:**
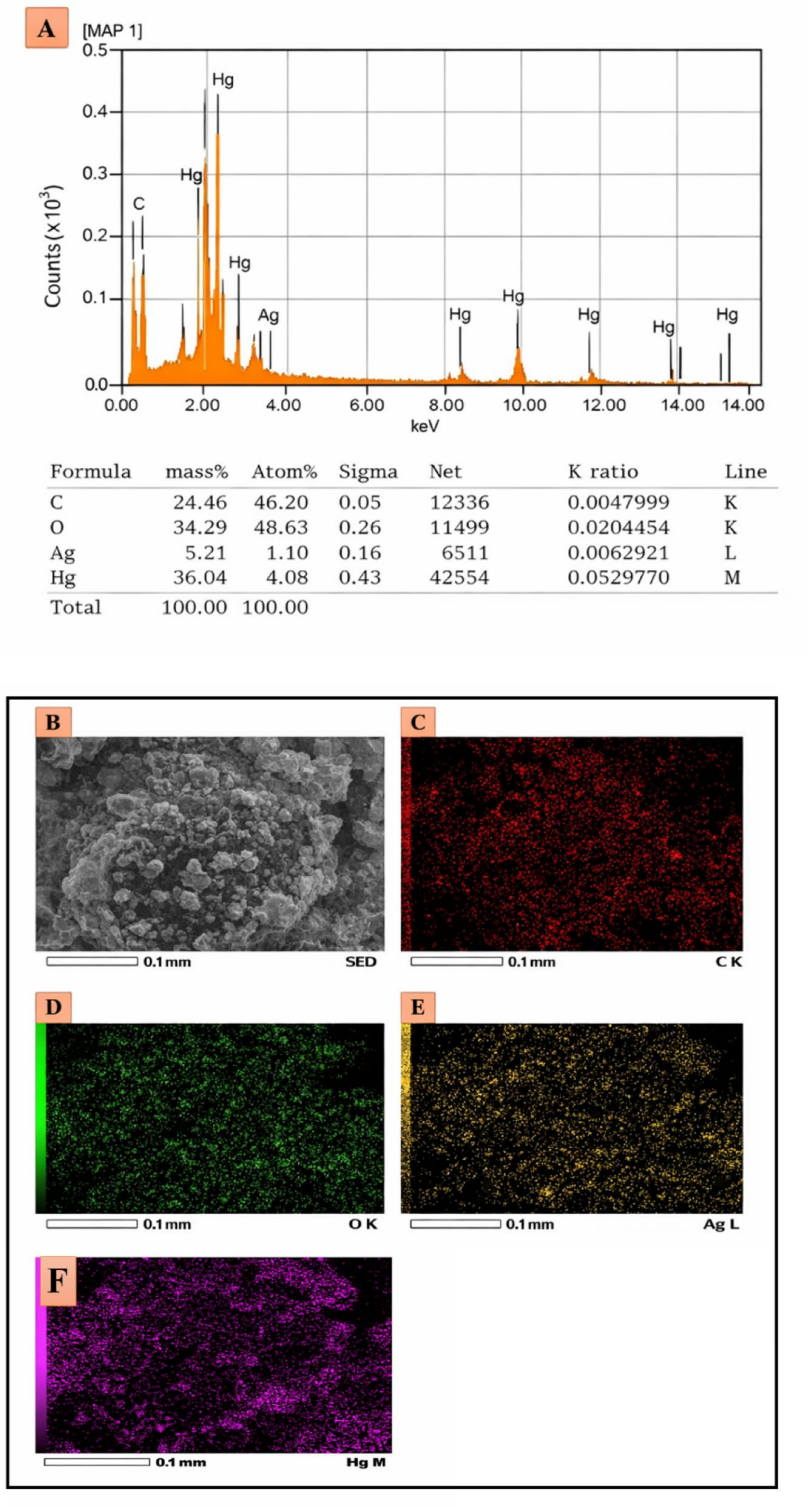
Elemental characterization of GW@AgNPs after interaction with Hg^2+^ ions. **A** EDX spectrum showing the presence of Ag and Hg. Elemental characterization of GW@AgNPs after interaction with Hg^2+^ ions. **B** SEM image, **C**–**F** Elemental mapping of carbon (C-K), oxygen (O-K), silver (Ag-L), and mercury (Hg-M), confirming distribution across the NPs surface

Elemental mapping and EDX analysis of the AgNPs synthesized using *G. wallichianum* extract and exposed to Hg^2+^ provided crucial insights into the interaction between the nanoparticles and mercury (Fig. 7A).

The SEM image provides a detailed view of the morphology of the AgNPs on the surface, indicating their shapes and distribution. The element maps show the spatial distribution of the most significant elements in the samples. The C K and O K maps confirmed the presence of carbon and oxygen, which may be attributed to the plant extract and oxidation of AgNPs, respectively. The presence of silver in the Ag L map indicates the successful synthesis of AgNPs, as shown in Fig. 7B. The Hg M map indicates the presence of mercury and the effective interaction between AgNPs and Hg^2+^ ions. This interaction is a major feature for the colorimetric detection of mercury as AgNPs alter color in the impact of mercury ions, enabling their use as environmental detectors. This is further evidenced by EDS analysis, which yields discrete peaks.

for carbon (C), oxygen (O), silver (Ag), and mercury (Hg), with the mercury peak indicating selective binding of the sample to Hg^2+^ ions. Elemental analysis provides both qualitative and quantitative data, showing the proportion of elements in the sample and the relative percentage ratio of silver and mercury. Mercury contamination detected in the EDX spectrum indicates that these green synthesized AgNPs can be used for sensitive and selective detection of mercury ions, which is valuable for environmental applications where mercury contamination is a concern.

### Analysis of Hg^2+^ in real water samples

Real water samples were analyzed to investigate the effectiveness of the newly developed sensor as an environmental monitoring device. The GW@AgNPs sensor was used to determine the amount of spiked Hg^2+^ in tap and river water, and the recovery rate was calculated. Three different concentrations (10, 20, and 40 µM) of Hg^2+^ were introduced into separate portions of tap and river water samples. The river water samples were collected from the Jehlum River in the Khushab district of Pakistan, and the tap water was collected from the Institute of Chemistry, University of Sargodha, Pakistan. Prior to spiking, the physicochemical properties of the tap and river water samples were measured to assess the matrix effects. The tap water had a pH of 7.4 and conductivity of 390 µS/cm, whereas the river water had a pH of 6.8 and conductivity of 570 µS/cm. Subsequently, the recovery of Hg^2+^ from the water samples was evaluated using the same analytical technique. The values for percentage recovery, mean standard deviation, standard error (SE) of intercept, standard deviation of intercept, relative standard deviation (% RSD), limit of determination (LOD), and limit of quantification (LOQ) for real water samples, with sample size of *n* = 3, are presented in Table 4. The results confirm that the GW@AgNPs nanoprobe, has the potential to be an effective and reliable sensor for measuring Hg^2+^ in real water samples. Even in the presence of natural ions and organics, the response curves for the GW@AgNPs sensor remained almost unchanged with low baseline drift, demonstrating good matrix effect tolerance. The % recovery values ranged from 88% to 99%, indicating that pH and conductivity did not obviously interfere with Hg^2+^ determination. The results validate the practical applicability of the sensor in real-world water samples without the need for complex pretreatment. However, further studies using heavily polluted or high-TDS water may be required to fully quantify matrix robustness.

**Table 4:** Colorimetric detection of Hg^2+^ in a real water sample

	Concentration (µM)	Found Hg^2+^ (µM)		% recovery		Found Hg^2+^ (µM)	% Recovery
River water	10		9.6	96	Tap water	8.8	88
	20		19.0	95		18.8	94
	40		39.8	99		39.4	98
	Average		96.7			Average	93.3
	Standard deviation		2.4			Standard deviation	5.3
	SE of intercept		0.003			SE of intercept	0.001
	SD of intercept		0.006			SD of intercept	0.002
	%RSD		2.5			%RSD	5.7
	LOD		3.3			LOD	1.0
	LOQ		11.1			LOQ	3.3

### Comparison with previous literature

The ecofriendly synthesis of AgNPs with *G. wallichianum* is a promising prospect in the field of plant-derived NPs preparation. The *G. wallichianum*-mediated AgNPs demonstrated broad-spectrum antibacterial *S. epidermidis*, *K. pneumoniae*, and *E. coli*. The antibacterial mechanisms, such as membrane perforation, ROS generation, silver ion release, and others, were found to be similar to those of other plant-based AgNPs reported in the literature, where NPs disrupt the structural integrity of bacterial cells, leading to inhibition.

The sunlight assisted green synthesis technique used in this study provides an eco-friendly and sustainable approach toward NPs production, while also minimizing costs by utilizing natural plant phytochemicals as both reducing agents and stabilizers. *G. wallichianum* serves a dual role in the synthesis of NPs and its application is more effective, than other conventional or chemically mediated methods.

Moreover, the ability of these AgNPs to selectively detect Hg^2+^ in aqueous solutions further demonstrates their versatility. The unique properties of *G. wallichianum*-mediated AgNPs, including their antibacterial activity and potential for environmental monitoring, make them promising materials for a wide range of biomedical and environmental applications. The present research adds to the increasing amount of literature on the sustainable multifunctional use of plant AgNPs and indicates their significance in managing antimicrobial resistance as well as environmental contamination. As shown in Table 5, the *G. wallichianum*-mediated AgNPs exhibit antibacterial activity comparable to AgNPs synthesized from other plants, underscoring their potential for wide-scale application.

**Table 5:** Comparative study with previously reported literature

Plant source (extract)	AgNP size (nm)	Target organisms	ZOI (mm)	Mechanism (summary)	References
*Azadirachta indica* (Neem) fruit pulp	30–40	Antibacterial activity (against common pathogens)	8–16	Guided by phytochemicals (flavonoids/terpenoids) reducing Ag⁺ to AgNP; release Ag⁺ interacts with bacterial membranes and proteins leading to cell death.	[108]
*A. indica* leaf	59–80	Against Multidrug-Resistant pathogens	8–12	Disruption of cell membrane, oxidative stress induction, protein binding.	[109]
*Curcuma longa* (Turmeric) flower	5	Against *Mycobacterium smegmatis*, *S. aureus*, *E. coli*	13–18	Small size enables penetration and membrane damage; release of silver ions disrupting cell wall and DNA function.	[110]
AgNPs – varied plants (general literature)	Typically 10–100	Multiple reports show significant activity depending on extract and bacteria tested	Varied ZOI	Antibacterial mechanisms include membrane disruption, ROS generation, metal ion release.	[111]
*Solanum tricobatum* and *Ocimum tenuiflorum*	*(size varies)*	Antibacterial activity against *S. aureus* and *E. coli*	7–13	Phytochemicals influence size/efficacy; smaller NPs often show stronger antibacterial action.	[112]
*Pandanus fascicularis*	25 nm	Antibacterial activity against *S. aureus* and *E. coli*	12–15	Bactericidal effects	[113]
*Fumaria indica*	40	Antibacterial activity (against common pathogens)	9–33	ROS generation, membrane disruption	[114]
*G. wallichianum*	29	*S. epidermidis*, *K. pneumoniae*,* E. coli*	18–20	ROS generation, membrane disruption, biomolecular damage (protein denaturation, DNA fragmentation)	This work

## Conclusions

This study successfully demonstrated the green synthesis of AgNPs using *G. wallichianum* leaves extract under sunlight exposure, providing a sustainable and cost-effective alternative to conventional methods. The development of AgNPs with a typical SPR peak at 425 nm was identified through UV-vis spectroscopy. The FTIR analysis showed that phytochemicals participated in the reduction and stabilization processes. XRD results showed that the AgNPs had a crystalline, face centered cubic structure, characterized by well-developed diffraction peaks of metallic silver. SEM analysis demonstrated a spherical morphology and an average particle size of 0.2583 nm, whereas DLS analysis demonstrated a hydrodynamic size of 0.39 nm, attributed to solvation layers and capping agents. Zeta potential analysis showed a surface charge of -29.3mV, indicating high colloidal stability. The EDX indicated the presence of elemental silver, with small amounts of carbon and oxygen detected due to plant biomolecules. The thermal stability and purity were proven using TGA/DSC thermograms. The biosynthesized GW@AgNPs exhibited strong antibacterial activity, with inhibition zones of 20, 18 and 19 mm against *S. epidermidis*,* K. pneumoniae*, and *E. coli* respectively. The MIC and MBC ranged from 15 to 30 µL (or µg/mL) and the statistical significance of differences between treatments was verified by ANOVA. The AgNPs also served as effective colorimetric sensors for Hg^2+^ detection with a detection limit of 6.68 µM, as well as high selectivity, making them useful for industrial and environmental mercury detection. This environmentally friendly synthesis method is scalable and inexpensive for biomedical and environmental applications. Nevertheless, further investigation of the biocompatibility and cytotoxicity of the synthesized AgNPs is necessary to determine their safe use in the medical field. Research on the stability and scalability of these AgNPs is advisable to determine their practical viability for large-scale environmental monitoring and industrial applications.

## Supplementary Information

Below is the link to the electronic supplementary material.

Supplementary Material 1

## Data Availability

All experimental supporting data and procedures are available within this article. The raw experimental data supporting the findings of this study, are available from the corresponding author upon reasonable request.

## References

[1] Prestinaci F, Pezzotti P, Pantosti A. Antimicrobial resistance: a global multifaceted phenomenon. Pathogens Global Health. Oct. 2015;109(7):309–18. 10.1179/2047773215Y.0000000030. 10.1179/2047773215Y.0000000030 PMC4768623 26343252

[2] Ibrahim SI, et al. Targeting ESKAPE pathogens with ZnS and Au@ ZnS Core–Shell nanoconjugates for improved biofilm control. Sci Rep. 2025;15(1):21407. 40595152 10.1038/s41598-025-07583-5 PMC12215030

[3] Dadgostar P. Antimicrobial Resistance: Implications and Costs, *IDR*, vol. Volume 12, pp. 3903–3910, Dec. 2019, 10.2147/IDR.S234610 10.2147/IDR.S234610 PMC6929930 31908502

[4] Parambil AM, Prasad A, Tomar AK, Ghosh I, Rajamani P. Biogenic carbon dots: a novel mechanistic approach to combat multidrug-resistant critical pathogens on the global priority list. J Mater Chem B. 2024;12(1):202–21. 10.1039/d3tb02374e 38073612

[5] Chandraker SK, Kumar R. Biogenic biocompatible silver nanoparticles: a promising antibacterial agent, *Biotechnology and Genetic Engineering Reviews*, vol. 40, no. 4, pp. 3113–3147, Nov. 2024, 10.1080/02648725.2022.2106084 10.1080/02648725.2022.2106084 35915981

[6] Paz S, et al. Toxic metals (Al, Cd, Pb and Hg) in the most consumed edible seaweeds in Europe. Chemosphere. 2019;218:879–84. 30609492 10.1016/j.chemosphere.2018.11.165

[7] Ehsan A, Sultana N, Munir B, Assad N, Ghaffar A, Naeem-ul-Hassan M. Assessment of some heavy metals for their potential health implications in the skin whitening creams available in Pakistani cosmetic market. Int J Environ Anal Chem. Sep. 2024;pp 1–13. 10.1080/03067319.2024.2408636.

[8] Khan MN et al. Cadmium dynamics: beneficial elements and chemical reactions in soil, in *Beneficial Elements for Remediation of Heavy Metals in Polluted Soil*, Elsevier, 2025, pp. 93–139. Accessed: Jul. 23, 2025. [Online].

[9] Suherman AL, Tanner EE, Compton RG. Recent developments in inorganic Hg^2+^ detection by voltammetry. TRAC Trends Anal Chem. 2017;94:161–72.

[10] Jabbar A, et al. A highly selective Hg^2+^ colorimetric sensor and antimicrobial agent based on green synthesized silver nanoparticles using *Equisetum diffusum* extract. RSC Adv. 2023;13(41):28666–75. 37790097 10.1039/d3ra05070j PMC10543206

[11] Hong-Xin N, et al. Clinicopathological features, diagnosis, and treatment of IgA nephropathy with minimal change disease related to exposure to mercury-containing cosmetics: a case report. Clin Nephrol. 2017;87(4):196. 28102816 10.5414/CN108967

[12] Kanwal M, et al. Dual colorimetric sensing of Hg (II) and Fe (III) using sulfanilamide-stabilized silver nanoparticles and evaluating their photodegradation and antibacterial properties. J Water Process Eng. 2025;75:107981.

[13] Chandraker SK, Lal M, Ghosh MK, Tiwari V, Ghorai TK, Shukla R. Green synthesis of copper nanoparticles using leaf extract of *Ageratum houstonianum* Mill. and study of their photocatalytic and antibacterial activities. Nano Express. 2020;1(1):010033.

[14] Mehra V, Kumar S, Tamang AM, Chandraker SK. Green synthesis of gold nanoparticles (AuNPs) by using plant extract and their biological application: A review. BioNanoScience. 2025;15(1):18.

[15] Ullah S et al. Phytomediated selenium doped Au/ZnO/Fe₃O₄ nanocomposites for synergistic photocatalytic dye degradation and Mercury (II) detection, *Scientific Reports*, 2025, Accessed: Dec. 11, 2025. [Online]. Available: https://www.nature.com/articles/s41598-025-30833-5 10.1038/s41598-025-30833-5 PMC12775441 41331318

[16] Khan I, Saeed K, Khan I. Nanoparticles: Properties, applications and toxicities. Arab J Chem. 2019;12(7):908–31.

[17] Velsankar K, Parvathy G, Sankaranarayanan K, Mohandoss S, Sudhahar S. Green synthesis of silver oxide nanoparticles using *Panicum miliaceum* grains extract for biological applications. Adv Powder Technol. 2022;33(7):103645.

[18] Velsankar K, Sudhahar S, Maheshwaran G. Effect of biosynthesis of ZnO nanoparticles via Cucurbita seed extract on *Culex tritaeniorhynchus* mosquito larvae with its biological applications. J Photochem Photobiol B. 2019;200:111650. 31698288 10.1016/j.jphotobiol.2019.111650

[19] Velsankar K, Parvathy G, Mohandoss S, Kumar RM, Sudhahar S. Green synthesis and characterization of CuO nanoparticles using *Panicum sumatrense* grains extract for biological applications. Appl Nanosci. 2022;12(6):1993–2021.

[20] Mehra V, Kumar S, Tamang AM, Chandraker SK. Green Synthesis of Gold Nanoparticles (AuNPs) by Using Plant Extract and Their Biological Application: A Review. BioNanoSci. Mar. 2025;15(1). 10.1007/s12668-024-01703-7.

[21] Ul Haq T, Ullah R, Khan MN, Nazish M, Almutairi SM, Rasheed RA. Seed priming with glutamic-acid-functionalized iron nanoparticles modulating response of *Vigna radiata* (L.) R. Wilczek (mung bean) to induce osmotic stress. Micromachines. 2023;14(4):736. 37420969 10.3390/mi14040736 PMC10141484

[22] Haq TU et al. Phyto-drug (Silymarin)-Encapsulated Cerium Oxide Nanoparticles (S-CeONPs) for *In-Vitro* Release, Ameliorating Antimicrobial, Anticancer, Anti-inflammatory and Antioxidant Potential, *BioNanoSci.*, Jan. 2024, 10.1007/s12668-023-01295-8

[23] Öztürk BY, Gürsu BY, Dağ İ. Antibiofilm and antimicrobial activities of green synthesized silver nanoparticles using marine red algae *Gelidium corneum*. Process Biochem. 2020;89:208–19.

[24] Alsharif SM et al. Multifunctional properties of spherical silver nanoparticles fabricated by different microbial taxa, *Heliyon*, vol. 6, no. 5, 2020, Accessed: Apr. 19, 2024. [Online]. Available: https://www.cell.com/heliyon/pdf/S2405-8440(20)30788-X.pdf 10.1016/j.heliyon.2020.e03943 PMC7268287 32518846

[25] Khan ZUR, et al. *Aconitum lycoctonum* L. (Ranunculaceae) mediated biogenic synthesis of silver nanoparticles as potential antioxidant, anti-inflammatory, antimicrobial and antidiabetic agents. BMC Chem. Sep. 2023;17(1):128. 10.1186/s13065-023-01047-5. 10.1186/s13065-023-01047-5 PMC10540474 37770921

[26] Hashmi SS, et al. Green synthesis of silver nanoparticles from *Olea europaea* L. extracted polysaccharides, characterization, and its assessment as an antimicrobial agent against multiple pathogenic microbes. Open Chem. Jan. 2024;22(1). 10.1515/chem-2024-0016.

[27] Assad N, et al. Nanotechnology in Oilseed Crops: A New Frontier for Abiotic Stress Adaptation. in Oilseed Crops Under Abiotic Stress: Mitigation Strategies and Future Perspectives. Springer; 2025. pp. 507–36.

[28] Waqar M et al. Green Synthesis of Silver Nanoparticles Using Chia (Salvia Hispanica L.) Seed Mucilage and Their Antioxidant, Antidiabetic, and Anti-Inflammatory Activities. Spectr Eng Sci, pp. 1929–44, 2025.

[29] Jacob JM, Ravindran R, Narayanan M, Samuel SM, Pugazhendhi A, Kumar G. Microalgae: A prospective low cost green alternative for nanoparticle synthesis. Curr Opin Environ Sci Health. 2021;20:100163.

[30] Assad N et al. Dec., Diffused sunlight assisted green synthesis of silver nanoparticles using *Cotoneaster nummularia* polar extract for antimicrobial and wound healing applications, *Natural Product Research*, pp. 1–15, 2023, 10.1080/14786419.2023.2295936 10.1080/14786419.2023.2295936 38146228

[31] Pugazhendhi A, Prabakar D, Jacob JM, Karuppusamy I, Saratale RG. Synthesis and characterization of silver nanoparticles using *Gelidium amansii* and its antimicrobial property against various pathogenic bacteria. Microb Pathog. 2018;114:41–5. 29146498 10.1016/j.micpath.2017.11.013

[32] Kováčová M, et al. Sustainable one-step solid-state synthesis of antibacterially active silver nanoparticles using mechanochemistry. Nanomaterials. 2020;10(11):2119. 33113789 10.3390/nano10112119 PMC7692266

[33] Hawar S N, Al-Shmgani H S., Al-Kubaisi A Z., Sulaiman M G., Dewir H Y., Rikisahedew J J. Green synthesis of silver nanoparticles from Alhagi graecorum leaf extract and evaluation of their cytotoxicity and antifungal activity. J Nanomaterials. 2022;2022:1–8.

[34] Shaheen S, Bibi M, Hussain H, Iqbal Saira I, Safdar Laraib S. A review on *Geranium wallichianum* D-Don ex-sweet: an endangered medicinal herb from Himalaya Region. Med Aromat Plants (Los Angles). 2017;6(288):1000288.

[35] Iqbal J, et al. Biogenic synthesis of green and cost effective cobalt oxide nanoparticles using *Geranium wallichianum* leaves extract and evaluation of *in vitro* antioxidant, antimicrobial, cytotoxic and enzyme inhibition properties. Mater Res Express. 2019;6(11):115407.

[36] De Silva GO, Abeysundara AT, Aponso MMW. Extraction methods, qualitative and quantitative techniques for screening of phytochemicals from plants. Am J Essent Oils Nat Prod. 2017;5(2):29–32.

[37] Assad N, et al. Eco-friendly synthesis of gold nanoparticles using *Equisetum diffusum* D. Don. with broad-spectrum antibacterial, anticancer, antidiabetic, and antioxidant potentials. Sci Rep. 2025;15(1):19246. 40456810 10.1038/s41598-025-02450-9 PMC12130309

[38] Assad N et al. Green Synthesis of Selenium Nanoparticles Using Equisetum diffusum: Characterization, Antibacterial Potential, Effects on Plant Growth, *IEEE Transactions on NanoBioscience*, 2025, Accessed: Jul. 23, 2025. [Online]. Available: https://ieeexplore.ieee.org/abstract/document/10988879/10.1109/TNB.2025.3567327 40327464

[39] Roheen T, et al. Enhanced Antibacterial and Anti-Inflammatory Efficiency of Serratiopeptidase Immobilized on CMC‐Silver Nanoparticles. J Basic Microbiol. May 2025. 10.1002/jobm.70060. 10.1002/jobm.70060 40401696

[40] Hsueh P-R, et al. Consensus statement on the adherence to Clinical and Laboratory Standards Institute (CLSI) Antimicrobial Susceptibility Testing Guidelines (CLSI-2010 and CLSI-2010-update) for Enterobacteriaceae in clinical microbiology laboratories in Taiwan. J Microbiol Immunol Infect. 2010;43(5):452–5. 21075714 10.1016/S1684-1182(10)60070-9

[41] Assad N, Abbas A, ur Rehman MF, Naeem-ul-Hassan M. Photo-catalytic and biological applications of phyto-functionalized zinc oxide nanoparticles synthesized using a polar extract of Equisetum diffusum D. RSC Adv. 2024;14(31):22344–58. 39010906 10.1039/d4ra03573a PMC11247436

[42] Kanwal M, et al. Dual colorimetric sensing of Hg (II) and Fe (III) using sulfanilamide-stabilized silver nanoparticles and evaluating their photodegradation and antibacterial properties. J Water Process Eng. 2025;75:107981.

[43] Mocak J, Bond AM, Mitchell S, Scollary G. A statistical overview of standard (IUPAC and ACS) and new procedures for determining the limits of detection and quantification: application to voltammetric and stripping techniques (technical report), *Pure and Applied Chemistry*, vol. 69, no. 2, pp. 297–328, 1997.

[44] Onyegbule F, Ilouno I, Ikeh C, Umeokoli B, Eze P. Evaluation of phytochemical constituents, analgesic, anti-inflammatory, antimicrobial and antioxidant activities of extracts of *Breynia nivosa* leaves. Planta Med. Oct. 2014;80(16):s–0034. 10.1055/s-0034-1395078.

[45] Ullah S, et al. Biosynthesis of phyto-functionalized silver nanoparticles using olive fruit extract and evaluation of their antibacterial and antioxidant properties. Front Chem. 2023;11:1202252. 37324561 10.3389/fchem.2023.1202252 PMC10262211

[46] Ajith MP, Pardhiya S, Prabhakar AK, Rajamani P. Ag@ CDs nanohybrid: Fabrication, design of a multi-mode chemosensory probe for selective Fe^3+^ detection and logic gate operation. Chemosphere. 2022;303:135090. 35660397 10.1016/j.chemosphere.2022.135090

[47] Abbas A, Amin HM. Silver nanoparticles modified electrodes for electroanalysis: An updated review and a perspective. Microchem J. 2022;175:107166.

[48] Das D, Haydar MS, Mandal P. Impact of physical attributes on proficient phytosynthesis of silver nanoparticles using extract of fresh mulberry leaves: characterization, stability and bioactivity assessment. J Inorg Organomet Polym Mater. 2021;31:1527–48.

[49] Alhomaidi E et al. Sep., Biosynthesis of silver nanoparticles using *Lawsonia inermis* and their biomedical application, *IET Nanobiotechnology*, vol. 16, no. 7–8, pp. 284–294, 2022, 10.1049/nbt2.12096 10.1049/nbt2.12096 PMC9469786 36039655

[50] Bukhari SNA et al. Hydroxypropylcellulose as a novel green reservoir for the synthesis, stabilization, and storage of silver nanoparticles, *IJN*, p. 2079, Mar. 2015. 10.2147/IJN.S75874 10.2147/IJN.S75874 PMC4368033 25844038

[51] Ullah I et al. Mar., *Peganum harmala* L. extract-based Gold (Au) and Silver (Ag) nanoparticles (NPs): Green synthesis, characterization, and assessment of antibacterial and antifungal properties, *Food Science & Nutrition*, p. fsn3.4112, 2024, 10.1002/fsn3.4112 10.1002/fsn3.4112 PMC11167161 38873463

[52] Gupta P, et al. Unveiling the cytotoxic and anti-proliferative potential of green-synthesized silver nanoparticles mediated by Colletotrichum gloeosporioides. RSC Adv. 2024;14(6):4074–88. 38292267 10.1039/d3ra06145k PMC10825743

[53] Irshad K, Akash MSH, Rehman K, Nadeem A, Shahzad A. Biosynthesis and Multifaceted Characterization of *Breynia nivosa* -Derived Silver Nanoparticles: An Eco-Friendly Approach for Biomedical Applications, *ACS Omega*, vol. 9, no. 13, pp. 15383–15400, Apr. 2024, 10.1021/acsomega.3c10119 10.1021/acsomega.3c10119 PMC10993374 38585127

[54] Aref MS, Salem SS. Bio-callus synthesis of silver nanoparticles, characterization, and antibacterial activities via *Cinnamomum camphora* callus culture. Biocatal Agric Biotechnol. 2020;27:101689.

[55] Smith BC. *Fundamentals of Fourier transform infrared spectroscopy*. CRC press, 2011. Accessed: Jan. 01, 2025. [Online]. Available: https://books.google.com/books?hl=en&lr=&id=LR9HkK2cP_0C&oi=fnd&pg=PP1&dq=+%22Infrared+Spectral+Interpretation.%22+CRC+Press+(2011).&ots=iNCcL2SJZw&sig=AXSbjZKnbPwIXo3NayKoqGI3kgo

[56] Bryce DL, Webster FX, Silverstein RM, Kiemle DJ. Spectrometric Identification of Organic Compounds. Wiley, 2014.

[57] Said A, Abu-Elghait M, Atta HM, Salem SS. Antibacterial Activity of Green Synthesized Silver Nanoparticles Using *Lawsonia inermis* Against Common Pathogens from Urinary Tract Infection. Appl Biochem Biotechnol. Jan. 2024;196(1):85–98. 10.1007/s12010-023-04482-1. 10.1007/s12010-023-04482-1 PMC10794286 37099124

[58] Abdellatif AAH, et al. Green Synthesis of Silver Nanoparticles Incorporated Aromatherapies Utilized for Their Antioxidant and Antimicrobial Activities against Some Clinical Bacterial Isolates. Bioinorg Chem Appl. Jan. 2022;2022(1):2432758. 10.1155/2022/2432758. 10.1155/2022/2432758 PMC9017581 35449714

[59] Pasieczna-Patkowska S, Cichy M, Flieger J. Application of Fourier transform infrared (FTIR) spectroscopy in characterization of green synthesized nanoparticles. Molecules. 2025;30(3):684. 39942788 10.3390/molecules30030684 PMC11821210

[60] Dogiparthi LK, et al. Phytochemical mediated synthesis of silver nanoparticles and their antibacterial activity. SN Appl Sci. Jun. 2021;3(6):631. 10.1007/s42452-021-04641-1.

[61] Jyoti K, Baunthiyal M, Singh A. Characterization of silver nanoparticles synthesized using *Urtica dioica* Linn. leaves and their synergistic effects with antibiotics. J Radiation Res and. 2016;9(3):217–27. Applied Sciences.

[62] Chinnathambi A, et al. Synthesis of AgNPs from leaf extract of *Naringi crenulata* and evaluation of its antibacterial activity against multidrug resistant bacteria. Environ Res. 2023;216:114455. 36202242 10.1016/j.envres.2022.114455

[63] Fitriany E, Priyoherianto A, Puspadina V, Arif MR, Alfulaila A, Shofiyyah MR. Green Synthesis AgNPs menggunakan Bioreduktor Alami Ekstrak Buah Kiwi: Biosintesis, dan Karakterisasi. Justek: Jurnal Sains dan Teknologi. 2023;6(1):162–9.

[64] Abdelmoneim HM, Taha TH, Elnouby MS, AbuShady HM. Extracellular biosynthesis, OVAT/statistical optimization, and characterization of silver nanoparticles (AgNPs) using *Leclercia adecarboxylata* THHM and its antimicrobial activity. Microb Cell Fact. Dec. 2022;21(1). 10.1186/s12934-022-01998-9. 10.1186/s12934-022-01998-9 PMC9801658 36581886

[65] Sadat A, Joye IJ. Peak fitting applied to fourier transform infrared and raman spectroscopic analysis of proteins. Appl Sci. 2020;10(17):5918.

[66] Han J-K, et al. Green synthesis of AgNPs using lignocellulose nanofibrils as a reducing and supporting agent. BioResources. 2020;15(2):2119.

[67] Md Salim R, Asik J, Sarjadi MS. Chemical functional groups of extractives, cellulose and lignin extracted from native *Leucaena leucocephala* bark, *Wood Sci Technol*, vol. 55, no. 2, pp. 295–313, Mar. 2021, 10.1007/s00226-020-01258-2

[68] Meng Y. A sustainable approach to fabricating Ag nanoparticles/PVA hybrid nanofiber and its catalytic activity. Nanomaterials. 2015;5(2):1124–35. 28347055 10.3390/nano5021124 PMC5312901

[69] Roy K, Sarkar CK, Ghosh CK. Green synthesis of silver nanoparticles using fruit extract of *Malus domestica* and study of its antimicrobial activity. Dig J Nanomater Biostruct. 2014;9(3):1137–47.

[70] Chandraker SK, Lal M, Kumar A, Shukla R. *Justicia adhatoda* L. mediated green synthesis of silver nanoparticles and assessment of their antioxidant, hydrogen peroxide sensing and optical properties, *Materials Technology*, vol. 37, no. 10, pp. 1355–1365, Aug. 2022, 10.1080/10667857.2021.1949525

[71] Daghian SG, Farahpour MR, Jafarirad S. Biological fabrication and electrostatic attractions of new layered silver/talc nanocomposite using *Lawsonia inermis* L. and its chitosan-capped inorganic/organic hybrid: investigation on acceleration of Staphylococcus aureus and Pseudomonas aeruginosa infected wound healing. Mater Sci Engineering: C. 2021;128:112294. 10.1016/j.msec.2021.112294 34474845

[72] Devatha CP, Thalla AK, Katte SY. Green synthesis of iron nanoparticles using different leaf extracts for treatment of domestic waste water. J Clean Prod. 2016;139:1425–35.

[73] Rocha V, Ferreira-Santos P, Aguiar C, Neves IC, Tavares T. Valorization of plant by-products in the biosynthesis of silver nanoparticles with antimicrobial and catalytic properties. Environ Sci Pollut Res. Jan. 2024;31(9):14191–207. 10.1007/s11356-024-32180-w. 10.1007/s11356-024-32180-w PMC10881659 38278998

[74] Rani P, et al. Highly stable AgNPs prepared via a novel green approach for catalytic and photocatalytic removal of biological and non-biological pollutants. Environ Int. 2020;143:105924. 32659527 10.1016/j.envint.2020.105924

[75] Alzubaidi AK, et al. Green synthesis and characterization of silver nanoparticles using flaxseed extract and evaluation of their antibacterial and antioxidant activities. Appl Sci. 2023;13(4):2182.

[76] Akhter MS et al. A systematic review on green synthesis of silver nanoparticles using plants extract and their bio-medical applications, *Heliyon*, 2024, Accessed: Nov. 19, 2024. [Online]. Available: https://www.cell.com/heliyon/fulltext/S2405-8440(24)05797-9 10.1016/j.heliyon.2024.e29766 PMC11140609 38828360

[77] Moond M, et al. Biosynthesis of silver nanoparticles utilizing leaf extract of *Trigonella foenum-graecum* L. for catalytic dyes degradation and colorimetric sensing of Fe^3+^/Hg^2+^. Molecules. 2023;28(3):951. 36770623 10.3390/molecules28030951 PMC9919385

[78] da Costa TS, da Silva MR, Barbosa JCJ, Neves UDSD, de Jesus MB, Tasic L. Biogenic silver nanoparticles’ antibacterial activity and cytotoxicity on human hepatocarcinoma cells (Huh-7). RSC Adv. 2024;14(4):2192–204. 38213978 10.1039/d3ra07733k PMC10777275

[79] Paosen S, Saising J, Septama AW, Voravuthikunchai SP. Green synthesis of silver nanoparticles using plants from Myrtaceae family and characterization of their antibacterial activity. Mater Lett. 2017;209:201–6.

[80] Sharafat U, et al. Biosynthesis of silver nanocatalyst and its application for the efficient hydrogenation of nitroarenes. Inorg Chem Commun. 2024;159:111667.

[81] Salam MA et al. Antimicrobial resistance: a growing serious threat for global public health, in *Healthcare*, MDPI, 2023, p. 1946. Accessed: Apr. 25, 2024. [Online]. Available: https://www.mdpi.com/2227-9032/11/13/1946 10.3390/healthcare11131946 PMC10340576 37444780

[82] Cohen ML. Changing patterns of infectious disease. Nature. 2000;406(6797):762–7. 10963605 10.1038/35021206

[83] Khan SN, Khan AU. Breaking the spell: combating multidrug resistant ‘superbugs’. Front Microbiol. 2016;7:174662. 10.3389/fmicb.2016.00174 PMC4757689 26925046

[84] Zheng K, Setyawati MI, Leong DT, Xie J. Antimicrobial silver nanomaterials. Coord Chem Rev. 2018;357:1–17.

[85] Bibi Y, et al. Phytosynthesis of gold nanoparticles from *Boerhavia diffusa* L. and their antibacterial, antifungal, antioxidant, and anticancer activities. Discover Nano. Jan. 2026;21(1):7. 10.1186/s11671-025-04425-1. 10.1186/s11671-025-04425-1 PMC12789338 41511634

[86] Gula H, Khan HA, Nasreen Z, Assad N, Turab SA, Hanif M. Exploring the Antibacterial, Anticoagulant, and Hemolytic Potential of Green-Synthesized Fe 2 O 3 Nanoparticles by Cucurbita pepo pulp, *IEEE Transactions on NanoBioscience*, 2025, Accessed: Jul. 23, 2025. [Online]. Available: https://ieeexplore.ieee.org/abstract/document/10973190/10.1109/TNB.2025.3563307 40261780

[87] Roheen T et al. Improved Destaining and Antimicrobial Potential of Pepsin Cross-linked HPMC-Se Nanoparticles, *IEEE Transactions on NanoBioscience*, 2025, Accessed: Jan. 30, 2026. [Online]. Available: https://ieeexplore.ieee.org/abstract/document/11316155/10.1109/TNB.2025.3648368 41452682

[88] Choi JS, et al. Antibacterial activity of green-synthesized silver nanoparticles using *Areca catechu* extract against antibiotic-resistant bacteria. Nanomaterials. 2021;11(1):205. 33466916 10.3390/nano11010205 PMC7830304

[89] Lara HH, Ayala-Núnez NV, L. del C. Ixtepan Turrent, and, Rodríguez C, Padilla. Bactericidal effect of silver nanoparticles against multidrug-resistant bacteria, *World Journal of Microbiology and Biotechnology*, vol. 26, pp. 615–621, 2010.

[90] Chandraker SK, et al. Bioengineered and biocompatible silver nanoparticles from Thalictrum foliolosum DC and their biomedical applications. Clean Technol Environ Policy. 2022;24(8):2479–94.

[91] Ouay BL, Stellacci F. Antibacterial activity of silver nanoparticles: A surface science insight. Nano Today. 2015;10(3):339–54.

[92] Chandraker SK, Kumar R. Biogenic biocompatible silver nanoparticles: a promising antibacterial agent, *Biotechnology and Genetic Engineering Reviews*, vol. 40, no. 4, pp. 3113–3147, Nov. 2024, 10.1080/02648725.2022.2106084 10.1080/02648725.2022.2106084 35915981

[93] Chandraker SK et al. Oct., Bioengineered and biocompatible silver nanoparticles from *Thalictrum foliolosum* DC and their biomedical applications, *Clean Techn Environ Policy*, vol. 24, no. 8, pp. 2479–2494, 2022, 10.1007/s10098-022-02329-7

[94] Xu L, Wang YY, Huang J, Chen CY, Wang ZX, Xie H. Silver nanoparticles: Synthesis, medical applications and biosafety. Theranostics. 2020;10(20):8996. 32802176 10.7150/thno.45413 PMC7415816

[95] Menichetti A, Mavridi-Printezi A, Mordini D, Montalti M. Effect of size, shape and surface functionalization on the antibacterial activity of silver nanoparticles. J Funct Biomaterials. 2023;14(5):244. 10.3390/jfb14050244 PMC10219039 37233354

[96] Kaur N, et al. Lycium shawii mediated green synthesis of silver nanoparticles, characterization and assessments of their phytochemical, antioxidant, antimicrobial properties. Inorg Chem Commun. 2024;159:111735.

[97] Ramalingam B, Parandhaman T, Das SK. Antibacterial Effects of Biosynthesized Silver Nanoparticles on Surface Ultrastructure and Nanomechanical Properties of Gram-Negative Bacteria viz. *Escherichia coli* and *Pseudomonas aeruginosa*, *ACS Appl. Mater. Interfaces*, vol. 8, no. 7, pp. 4963–4976, Feb. 2016, 10.1021/acsami.6b00161 10.1021/acsami.6b00161 26829373

[98] Noor A et al. Apr., Green Synthesis of Silver-Doped ZnO Nanoparticles From *Adiantum venustum* D. Don (Pteridaceae): Antimicrobial and Antioxidant Evaluation, *Journal of Basic Microbiology*, vol. 65, no. 4, p. e2400543, 2025, 10.1002/jobm.202400543 10.1002/jobm.202400543 39807572

[99] Geremew A, et al. Green Synthesis of Novel Silver Nanoparticles Using *Salvia blepharophylla* and *Salvia greggii*: Antioxidant and Antidiabetic Potential and Effect on Foodborne Bacterial Pathogens. Int J Mol Sci. 2024;25(2):904. 38255978 10.3390/ijms25020904 PMC10815671

[100] Yousaf H, Mehmood A, Ahmad KS, Raffi M. Green synthesis of silver nanoparticles and their applications as an alternative antibacterial and antioxidant agents. Mater Sci Engineering: C. 2020;112:110901. 10.1016/j.msec.2020.110901 32409057

[101] Ghaffar N, et al. Metal nanoparticles assisted revival of Streptomycin against MDRS *Staphylococcus aureus*. PLoS ONE. 2022;17(3):e0264588. 35324924 10.1371/journal.pone.0264588 PMC8947119

[102] Chandraker SK, Lal M, Shukla R. DNA-binding, antioxidant, H 2 O 2 sensing and photocatalytic properties of biogenic silver nanoparticles using *Ageratum conyzoides* L. leaf extract. RSC Adv. 2019;9(40):23408–17. 35514502 10.1039/c9ra03590g PMC9067290

[103] Chandraker SK, Lal M, Dhruve P, Singh RP, Shukla R. Cytotoxic, antimitotic, DNA binding, photocatalytic, H2O2 sensing, and antioxidant properties of biofabricated silver nanoparticles using leaf extract of *Bryophyllum pinnatum* (Lam.) Oken. Front Mol Biosci. 2021;7:593040. 33585553 10.3389/fmolb.2020.593040 PMC7876318

[104] Chandraker SK, Ghosh MK, Lal M, Ghorai TK, Shukla R. Colorimetric sensing of Fe ^3+^ and Hg ^2+^ and photocatalytic activity of green synthesized silver nanoparticles from the leaf extract of *Sonchus arvensis* L. New J Chem. 2019;43(46):18175–83.

[105] Tooba, et al. Green synthesis of silver nanoparticles using *Solanum lycopersicum* leaves extract for highly selective detection of mercury ions and photocatalytic degradation of methylene blue. Discover Nano. Jan. 2026;21(1):14. 10.1186/s11671-025-04424-2. 10.1186/s11671-025-04424-2 PMC12816498 41553636

[106] Jain S, Nehra M, Dilbaghi N, Chaudhary GR, Kumar S. Detection of Hg^2+^ Using a Dual-Mode Biosensing Probe Constructed Using Ratiometric Fluorescent Copper Nanoclusters@Zirconia Metal-Organic Framework/ *N* -Methyl Mesoporphyrin IX and Colorimetry G-Quadruplex/Hemin Peroxidase-Mimicking G-Quadruplex DNAzyme. BME Front. Jan. 2024;5:0078. 10.34133/bmef.0078. 10.34133/bmef.0078 PMC11650877 39691776

[107] Shiva Prasad K, Shruthi G, Shivamallu C. Functionalized silver nano-sensor for colorimetric detection of Hg^2+^ ions: facile synthesis and docking studies. Sensors. 2018;18(8):2698. 30115894 10.3390/s18082698 PMC6111407

[108] Babu AB, Rao YS, Sravanthi P, Miditana SR, Pulsingh D, Bhavani AD. Green synthesis of silver nanoparticles using *Azadirachta indica* (Neem) fruit pulp extract and their antioxidant, antibacterial, and anticancer activity. Sustainable Chem Environ, p. 100282, 2025.

[109] Alqahtani YS, et al. In vitro antibacterial activity of green synthesized silver nanoparticles using *Mangifera indica* aqueous leaf extract against multidrug-resistant pathogens. Antibiotics. 2022;11(11):1503. 36358157 10.3390/antibiotics11111503 PMC9686697

[110] Rajak KK, Pahilani P, Patel H, Kikani B, Desai R, Kumar H. Green synthesis of silver nanoparticles using *Curcuma longa* flower extract and antibacterial activity, Apr. 10, 2023, *arXiv*: arXiv:2304.04777. 10.48550/arXiv.2304.04777

[111] Vanlalveni C, Lallianrawna S, Biswas A, Selvaraj M, Changmai B, Rokhum SL. Green synthesis of silver nanoparticles using plant extracts and their antimicrobial activities: A review of recent literature. RSC Adv. 2021;11(5):2804–37. 35424248 10.1039/d0ra09941d PMC8694026

[112] Eker F, Akdaşçi E, Duman H, Bechelany M, Karav S. Green synthesis of silver nanoparticles using plant extracts: A comprehensive review of physicochemical properties and multifunctional applications. Int J Mol Sci. 2025;26(13):6222. 40650001 10.3390/ijms26136222 PMC12250056

[113] Bedoura S, Habibullah ABM, Islam MS, Mahin MMA, Anwar MT, Shoily MT. Green synthesis and molecular-level mechanism of silver nanoparticles from *Pandanus fascicularis* (Keya) leaf extract with antibacterial activity. Next Nanatechnol. 2026;9:100376.

[114] Rahman A, et al. Phytomediated synthesis of silver nanoparticles using *Fumaria indica* extract with antibacterial, antifungal and antioxidant properties. Discov Sustain. Mar. 2026;7(1):399. 10.1007/s43621-026-02837-2.

