# Supplementary material for: Synthesis of bio-inspired silver nanoparticles using Geranium wallichianum D. Don ex Sweet. (Geraniaceae) leaf extract for antibacterial activity and colorimetric detection of Hg2+
Source: Discov Nano. 2026 May 29;21(1):231. doi: 10.1186/s11671-026-04673-9 (PMC13221511; doi:10.1186/s11671-026-04673-9)

**Synthesis of Bio-inspired Silver Nanoparticles using *Geranium wallichianum* Leaf Extract for Antibacterial Activity and Colorimetric Detection of Hg²⁺**

Attia Khalid^1^, Nasir Assad^1^, Muhammad Naeem-ul-Hassan^1^, Marzia Batool Laila^1^, Dalya Marwan Attallah^2^, Shadi A. Zakai^3^, Khalil Alkuwaity^4,5^, Yasir Assad^6^, Rao Muhammad Faisal Iqbal^7^, Muhammad Nauman Khan^8^, Alevcan Kaplan^9^, Majid Khan^10^, Shabab Hussain^11*^

1. Institute of Chemistry, University of Sargodha, Sargodha 40100, Pakistan, attiakhalid555@gmail.com (AK); [nakhan_98@yahoo.com](mailto:nakhan_98@yahoo.com) (NA); sobheel@yahoo.com (MNUH); [marziabatoollaila@gmail.com](mailto:marziabatoollaila@gmail.com) (MBL)
2. Department of Clinical Microbiology Laboratory, King Abdulaziz University Hospital, Jeddah, Saudi Arabia, datallha@kau.edu.sa (DMA)
3. Department of Clinical Microbiology and Immunology, Faculty of Medicine, King Abdulaziz University, Jeddah, 21589, Saudi Arabia, szakai@kau.edu.sa (SAZ)
4. Department of Medical Laboratory Sciences, Faculty of Applied Medical Sciences, King Abdulaziz University, Jeddah 21589, Saudi Arabia, kalkuwaity@kau.edu.sa (KA)
5. EcoHealth Unit, King Fahd Medical Research Center, King Abdulaziz University, Jeddah 21589, Saudi Arabia.
6. Department of Zoology, Hazara University Mansehra, Mansehra, Khyber Pakhtunkhwa, Pakistan, [yasirassad01@gmail.com](mailto:yasirassad01@gmail.com) (YA)
7. Department of Chemistry, The University of Lahore, Sargodha Campus, 10 km Lahore road, Sargodha, Pakistan, faisaliqbal1740@gmail.com (RMFI)
8. Department of Botany, Islamia College Peshawar, 25120 Peshawar, Pakistan; [nomiflora@uop.edu.pk](mailto:nomiflora@uop.edu.pk) (MNK)
9. Department of Pharmaceutical Botany, Faculty of Pharmacy, Dicle University, 21200 Diyarbakır, Turkey; kaplanalevcan@gmail.com (AK)
10. Institute of Biotechnology and Genetic Engineering, The University of Agriculture Peshawar, Peshawar 25130, Pakistan; m.khan@aup.edu.pk (MK)
11. Department of Biomedical and Clinical Sciences, University of Milan, 20157, Via Festa del Perdono, Milano, Italy; shabab.hussain@unimi.it (SH)

**Corresponding author**: shabab.hussain@unimi.it (SH)

**Table 1S.** Phytochemicals analysis of the polar extract of *G. wallichianum* carried out in triplicate*.* (Symbols: (+) indicates a positive result, (-) indicates a negative result)

| **Test** | **Result** |
| --- | --- |
| Phenol | + |
| Coumarins | - |
| Lignins | - |
| Faponins | + |
| Flavonoids | + |
| Alkaloids | + |
| Sterols | - |
| Leucoanthocyanin | - |
| Glycosides | + |

**Table 2S:** One-way ANOVA results showing statistically significant differences (p < 0.05) in antibacterial activity (zone of inhibition) among treatments (AgNPs, plant extract, and control) against selected bacterial strains*.*

| **Bacterial Strain** | **F-Statistic** | **p-Value** | **Significance** |
| --- | --- | --- | --- |
| *S. epidermidis* | 715.75 | 7.27 × 10⁻⁸ | Significant |
| *K. pneumonia* | 537.25 | 1.71 × 10⁻⁷ | Significant |
| *E. coli* | 643.00 | 1.00 × 10⁻⁷ | Significant |

**Table 3S.** One-way ANOVA results for MIC values of AgNPs against different bacterial strains, indicating statistically significant differences (p < 0.05) in antimicrobial effectiveness between treatments.

| **Bacterial Strain** | **F-Statistic** | **p-Value** | **Interpretation** |
| --- | --- | --- | --- |
| *S. epidermidis* | 420.25 | 3.34 × 10⁻^5^ | Significant difference between treatments (p < 0.05) |
| *K. pneumonia* | 529.00 | 2.12 × 10⁻^5^ | Significant difference between treatments (p < 0.05) |
| *E. coli* | 870.25 | 7.86 × 10⁻^6^ | Significant difference between treatments (p < 0.05) |

**Table 4S.** One-way ANOVA results for MBC values of AgNPs against different bacterial strains, indicating statistically significant differences (p < 0.05) in antimicrobial effectiveness between treatments.

| **Bacterial Strain** | **F-Statistic** | **p-Value** | **Interpretation** |
| --- | --- | --- | --- |
| *S. epidermidis* | 484.00 | 2.53 × 10⁻^5^ | Significant difference between treatments (p < 0.05) |
| *K. pneumonia* | 462.25 | 2.77 × 10⁻^5^ | Significant difference between treatments (p < 0.05) |
| *E. coli* | 1369.00 | 3.19 × 10⁻^6^ | Significant difference between treatments (p < 0.05) |

**Figure 1S.** TGA/DSC thermogram of green synthesized AgNPs showing high thermal stability with minimal weight loss up to 600 °C and a significant residue of 70.47% at 818 °C, confirming the formation of stable metallic silver nanoparticles. The sharp endothermic peak at 653.01 °C corresponds to the thermal behavior of AgNPs, with no decomposition event indicative of AgO presence.


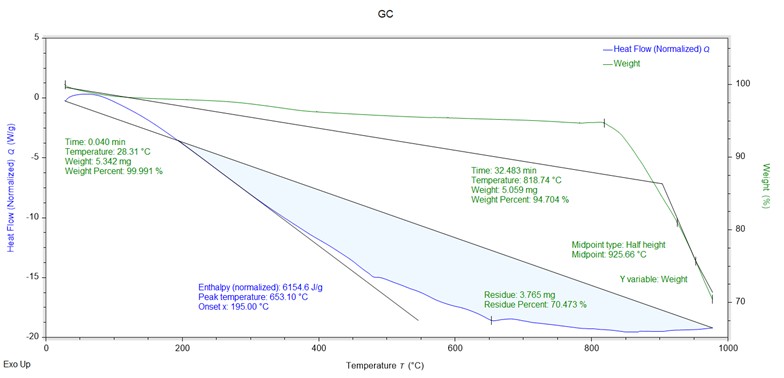

Supplement: Supplementary file 1 — Supplementary Material 1 [file 11671_2026_4673_MOESM1_ESM.docx]
